# Unveiling the Anticancer Potential: Computational Exploration of Nitrogenated Derivatives of (+)-Pancratistatin as Topoisomerase I Inhibitors

**DOI:** 10.3390/ijms251910779

**Published:** 2024-10-07

**Authors:** Magdi Awadalla Mohamed, Tilal Elsaman, Abozer Y. Elderdery, Abdullah Alsrhani, Heba Bassiony Ghanem, Majed Mowanes Alruwaili, Siddiqa M. A. Hamza, Salma Elhadi Ibrahim Mekki, Hazim Abdullah Alotaibi, Jeremy Mills

**Affiliations:** 1 Department of Pharmaceutical Chemistry, College of Pharmacy, Jouf University, Sakaka 72388, Saudi Arabia; 2 Department of Clinical Laboratory Sciences, College of Applied Medical Sciences, Jouf University, Sakaka 42421, Saudi Arabia; ayelderdery@ju.edu.sa (A.Y.E.); afalserhani@ju.edu.sa (A.A.); hbghanem@ju.edu.sa (H.B.G.); 3 Nursing Administration & Education Department, College of Nursing, Jouf University, Sakaka 72388, Saudi Arabia; majed@ju.edu.sa; 4 Department of Pathology, College of Medicine, Umm Alqura University, Algunfudah 21912, Saudi Arabia; smhamza@uqu.edu.sa; 5 Deparment of Physiology, College of Medicine, Umm Alqura University, Alqunfudah 21912, Saudi Arabia; semekki@uqu.edu.sa; 6 Department of Medical Oncology, Prince Mohammad Medical City, Aljouf 11443, Saudi Arabia; hazim111@hotmail.com; 7 School of Medicine, Pharmacy and Biomedical Sciences, University of Portsmouth, Portsmouth PO1 2DT, UK; jeremy.mills@port.ac.uk

**Keywords:** cancer, Topoisomerase I, CADD, (+)-Pancratistatin, *Amaryllidaceae* alkaloids

## Abstract

Cancer poses a substantial global health challenge, driving the need for innovative therapeutic solutions that offer improved effectiveness and fewer side effects. Topoisomerase I (Topo I) has emerged as a validated molecular target in the pursuit of developing anticancer drugs due to its critical role in DNA replication and transcription. (+)-Pancratistatin (PST), a naturally occurring compound found in various *Amaryllidaceae* plants, exhibits promising anticancer properties by inhibiting Topo I activity. However, its clinical utility is hindered by issues related to limited chemical availability and aqueous solubility. To address these challenges, molecular modelling techniques, including virtual screening, molecular docking, molecular mechanics with generalised born and surface area solvation (MM-GBSA) calculations, and molecular dynamics simulations were utilised to evaluate the binding interactions and energetics of PST analogues with Topo I, comparing them with the well-known Topo I inhibitor, Camptothecin. Among the compounds screened for this study, nitrogenated analogues emerged as the most encouraging drug candidates, exhibiting improved binding affinities, favourable interactions with the active site of Topo I, and stability of the protein-ligand complex. Structural analysis pinpointed key molecular determinants responsible for the heightened potency of nitrogenated analogues, shedding light on essential structural modifications for increased activity. Moreover, in silico absorption, distribution, metabolism, excretion, and toxicity (ADMET) predictions highlighted favourable drug-like properties and reduced toxicity profiles for the most prominent nitrogenated analogues, further supporting their potential as effective anticancer agents. In summary, this screening study underscores the significance of nitrogenation in augmenting the anticancer efficacy of PST analogues targeting Topo I. The identified lead compounds exhibit significant potential for subsequent experimental validation and optimisation, thus facilitating the development of novel and efficacious anticancer therapeutics with enhanced pharmacological profiles.

## 1. Introduction

**Figure 1:**
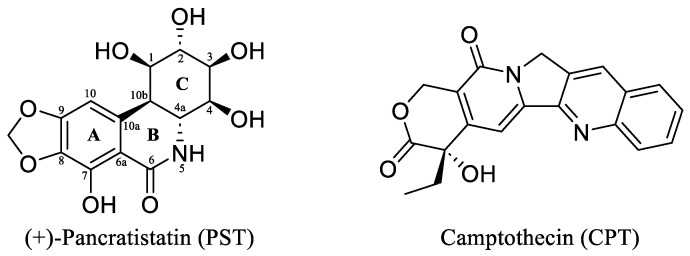
The chemical structures of the *Amaryllidaceae* alkaloid (+)-Pancratistatin (PST) and the Topo I inhibitor Camptothecin (CPT).

**Figure 2:**
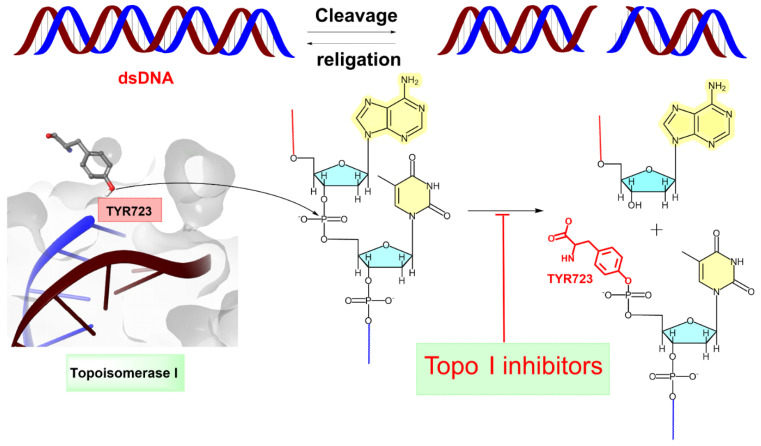
Topoisomerase I (Topo I) catalyses the transient breakage of DNA strands. This process involves a transesterification reaction where the enzyme tyrosyl group “TYR 723” forms a covalent bond with the DNA phosphate group, resulting in the breakage of the DNA backbone. The bond is later rejoined by reversing this reaction, restoring the DNA structure [26]. The blue line indicates the linkage between the 3′-phosphate group of one nucleotide and the 5′-OH group of the adjacent nucleotide. The red line represents the attachment between the 5′-OH group of one nucleotide and the 3′-phosphate group of the neighboring nucleotide.

Cancer is a complex disease characterised by abnormal cell growth and spread, posing challenges for patients and healthcare providers [1]. Causes include genetic, environmental, and lifestyle factors, leading to over 100 types of cancer, each requiring individual treatment approaches for the best outcomes [2,3]. Common treatments include surgery, chemotherapy, and radiation therapy, with newer options like immunotherapy and targeted therapy [4]. Early detection and treatment are vital for improving outcomes [5]. Challenges in cancer treatment include its complexity, resistance, recurrence, metastasis, and medication side effects, highlighting the urgent need for new drugs to improve treatment, save lives, and reduce the global burden of cancer [6,7]. Drug discovery is essential for medical progress, especially in cancer treatment, and researchers worldwide are committed to finding new therapeutic targets, creating innovative treatments, and improving current therapies for cancer [8,9]. Natural products sourced from plants, animals, fungi, or microorganisms with traditional medicinal uses offer valuable potential for anticancer drugs [10]. Ongoing research aims to fully utilise natural products, opening avenues for novel anticancer agents and therapeutic approaches [9]. Computer-Aided Drug Design (CADD) is now pivotal in cancer research and drug discovery, utilising computational methods to hasten the identification, design, and enhancement of novel anticancer compounds [11]. Through these techniques, researchers streamline the process, reducing the time, costs, and resources required for wet lab validation [12].

(+)-Pancratistatin (PST), a phenanthridone alkaloid (Figure 1) naturally occurring in *Amaryllidaceae* plants, has attracted considerable attention due to its potent anticancer properties and distinctive mechanisms of action [13,14,15]. Studies have shown that PST exhibits inhibitory activity against Topoisomerase I (Topo I), a significant target for anticancer drugs, though it is activity is weaker when compared to the well-known inhibitor Camptothecin (CPT) (Figure 1) [16]. Topo I plays a crucial role in DNA metabolism, including replication, transcription, and repair. Inhibiting this enzyme can result in DNA damage and subsequent cancer cell death [17]. TYR 723 is a critical residue within the active site of the enzyme [18]. It plays a central role in the catalytic mechanism of Topo I and is involved in the nucleophilic attack on the phosphodiester backbone of the DNA strand during the cleavage and religation steps of the enzyme’s catalytic cycle. During the cleavage reaction, Topo I forms a covalent intermediate with the 3′ end of the DNA strand by forming a phosphotyrosine bond between the TYR 723 of the enzyme and the phosphate group of the DNA backbone (Figure 2). This covalent complex allows the enzyme to break one strand of the DNA double helix, relieving torsional strain. After DNA strand rotation or relaxation occurs, relegation of the DNA strand takes place. TYR 723 facilitates religation by attacking the 5′ end of the broken DNA strand, resulting in the release of the enzyme from the DNA and the sealing of the DNA break. Besides TYR 723, numerous other amino acid residues within the active site of Topo I play crucial roles in its catalytic activity. These residues encompass ASN 722, LYS 532, ASP 533, ARG 364 and ASN 352, each contributing to different stages of the enzyme’s catalytic cycle, including DNA binding, cleavage and religation [19,20,21,22,23,24,25,26].

Derivatives of PST have been synthesised to improve its pharmacological properties and efficacy and address challenges like low yield and poor bioavailability. Derivatisation involves chemically modifying PST’s structure to enhance its stability, solubility, and target specificity while preserving or enhancing its anticancer activity [27,28]. The current study aims to computationally explore the potential of PST and its derivatives in inhibiting Topo I activity using the well-known inhibitor CPT as a standard.

## 2. Results and Discussion

**Figure 3:**
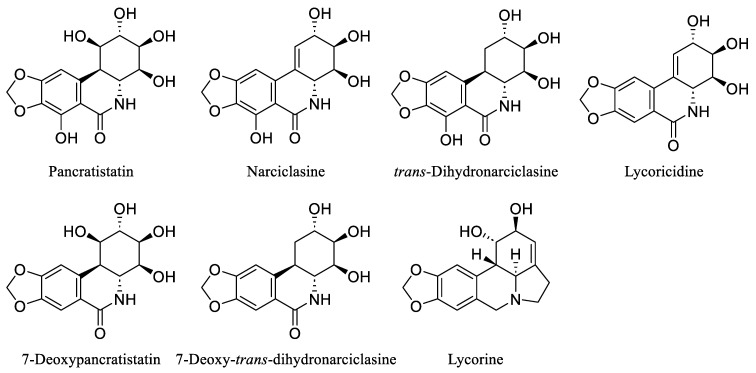
Chemical structures of some natural *Amaryllidaceae* alkaloids.

Over the last few decades, the investigation of natural products in both marine and terrestrial environments has led to the discovery of many biologically active alkaloids. Within this category are the *Amaryllidaceae* alkaloids, a set of structurally related compounds primarily sourced from plants within the *Amaryllidaceae* family, including Pancratistatin, Narciclasine, *trans*-Dihydronarciclasine, Lycoricidine, 7-Deoxypancratistatin, 7-Deoxy-*trans*-dihydronarciclasine, and Lycorine [29,30,31] (Figure 3).

The renowned *Amaryllidaceae* alkaloid PST is recognised for its highly oxygenated phenanthridinone core consisting of rings A, B, and C, with ring C featuring successive chiral centres (Figure 1). PST was initially isolated in 1984 by Pettit et al. from the roots of Hawaiian *Pancratium littorale* [32]. Its intricate structure and reported in vitro anticancer properties have captured the interest of medicinal chemists. Nonetheless, the preclinical assessment of PST has been put on hold due to the limited availability of the isolated material. As a result, there has been significant interest regarding the total synthesis of PST to enable further preclinical investigations. During the last forty years, several synthetic studies have been undertaken to address this goal [33,34,35]. Despite significant efforts from many researchers, the synthesis of PST has often been limited to the milligram scale, thus hindering further exploration of its pharmacological and clinical properties. In 2022, Ding et al. reported an efficient gram-scale synthesis of PST, facilitating comprehensive biological testing of its anticancer properties [16]. It was revealed, for the first time, that PST has the potential to act as a Topoisomerase I (Topo I) inhibitor, particularly when compared to CPT as a known specific inhibitor of the enzyme. However, PST exhibited a weaker activity (2-fold lower) in inhibiting the conversion of supercoiled DNA to relaxed DNA compared to CPT. Hence, the present study endeavoured to identify potent analogues of PST with Topo I inhibitory activity using a computational approach.

### 2.1. Molecular Docking Protocol Validation

Multiple X-ray crystal structures of Topo I are available in the Protein Data Bank (www.rcsb.org, accessed on 10 March 2023), identified by unique PDB codes (e.g., 1T8I, 1K4T, 1SEU, and 1SC7). Only protein structures crystallised with CPT as the inhibitor were examined, as employed by Ding et al. [16], as the reference standard to assess the potential of PST as a Topo I inhibitor. For docking protocol validation, the co-crystallized ligand, CPT, was redocked with the corresponding protein. Crystal structure 1T8I emerged as the optimal selection, with its co-crystallized ligand, CPT, demonstrating the most favourable RMSD value of 0.39 [36]. Consequently, the decision was made to proceed with the crystal structure 1T8I for further analysis.

**Figure 4:**
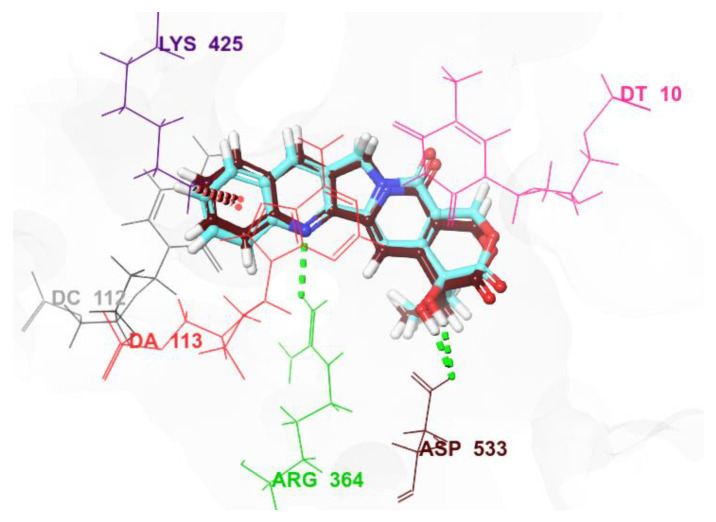
A superimposition of the docked co-crystallized ligand CPT (in cyan) with the original ligand (in brown).

Molecular docking is a valuable computational tool that enhances experimental methods in drug discovery and structural biology. It facilitates quicker and more cost-efficient identification and refinement of potential drug candidates [37]. For the effective evaluation of binding site accuracy and the improvement of reliability in docking studies within drug discovery and structural biology, it is imperative that docking protocols undergo validation and refinement [38]. In this context, redocking CPT onto the protein (PDB ID: 1T8I), prepared using “protein preparation wizard” [39], provided compelling evidence for the reliability and accuracy of the docking protocol. CPT seamlessly bound within the 1T8I binding pocket, displaying a commendably low RMSD value of 0.39 (Figure 4). The corresponding docking score and MM-GBSA energy were determined as −8.87 kcal/mol and −60.69 kcal/mol, respectively (Table 1).

### 2.2. Molecular XP Docking

**Figure 5:**
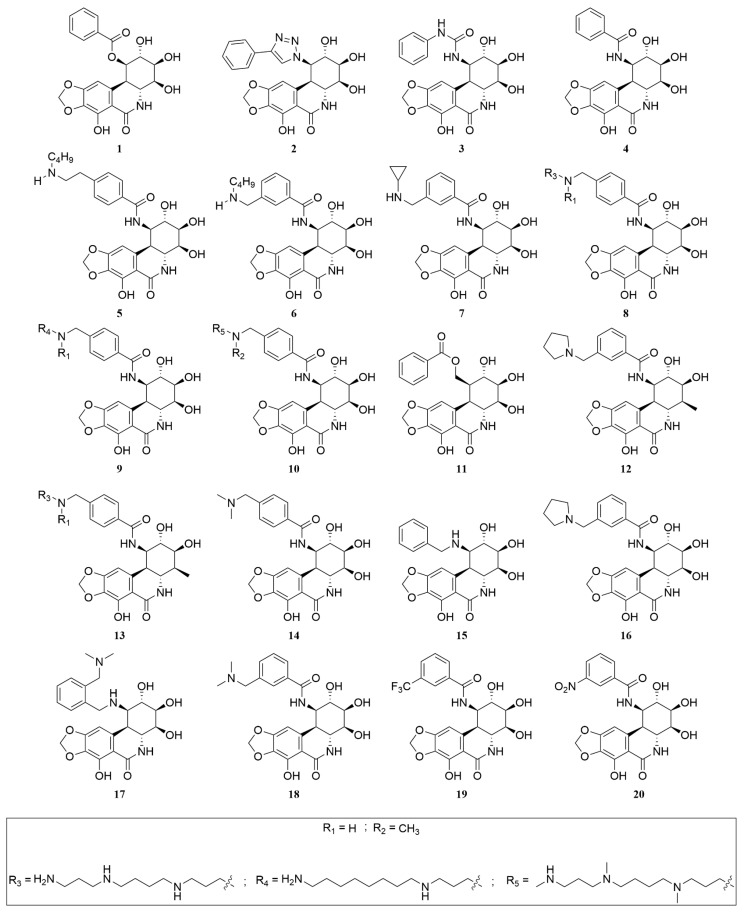
Chemical structures of PST analogues.

A collection of 440 analogues of PST was assembled from sources including PubChem and the published literature [40]. Each analogue, along with PST and CPT, underwent processing using the “ligprep” tool (Schrödinger Release 2023-2: LigPrep, Schrödinger, LLC, New York, NY, USA, 2023) before being subjected to extra precision (XP) docking, utilising the established docking protocol. While PST yielded a modest docking score of −7.10 kcal/mol in comparison to CPT’s −8.87 kcal/mol, 13 analogues (**2**, **5–10**, **12–14**, **16**, **18** and **20**) (Figure 5) with similar stereochemical configuration of PST demonstrated equal or superior docking scores ranging from −8.88 to −15.79 kcal/mol when compared to CPT’s score (Table 1).

PST displayed distinct binding behaviour compared to CPT when bound to 1T8I. It formed hydrogen bonds with three residues: ASN 352 (2.21Å), TYR 426 (1.85Å and 2.03Å), and MET 428 (1.94Å) (Supplementary Table S1). It did not exhibit Pi-Pi stacking interactions with either the protein or the DNA. The tested analogues of PST displayed varied binding modes contingent upon the functional groups they contained. Nevertheless, a common binding mode emerged wherein all analogues assumed an intercalating position, engaging in diverse binding interactions, such as hydrogen bonds, salt bridges, Pi-Pi stacking, and Pi-cation interactions. For example, compound **8a**, which ranked highest in terms of docking score, intercalated between the base pairs of the DNA, establishing both Pi-Pi stacking and Pi-cation interactions with DT 10 (4.31Å and 4.30Å, respectively). Additionally, it formed a hydrogen bond with DA 13 (2.51Å) and two hydrogen bonds with DA 113 (1.75Å and 1.94Å). It made two contacts with ASP 533, one through hydrogen bonding (2.08Å) and the other via a salt bridge (3.01Å) (Supplementary Table S1). It is noteworthy that ASP 533 is a critical residue involved in the catalytic function of Topo I. Furthermore, it has been demonstrated that this residue enhances the enzyme’s sensitivity to the bound inhibitor [18]. Compound **8a** established hydrogen bonds not only with the ASP 533 residue of 1T8I but also with ASN 352 (2.12Å), another crucial residue [41] and TYR 426 (2.40Å).

### 2.3. MM-GBSA Binding Free Energy Calculations

Molecular docking typically provides information about the potential binding poses of ligands within a target protein’s binding site [37]. While docking can predict binding modes reasonably well, it often struggles with accurately predicting binding affinities [42]. Molecular mechanics with generalised born and surface area solvation (MM-GBSA), on the other hand, is a post-docking method used to refine and improve the binding affinity predictions obtained from docking studies [43]. In this study, MM-GBSA calculations were conducted using the complexes obtained from XP docking. The co-crystallized ligand CPT demonstrated an approximately 2-fold higher affinity for Topo I (PDB: 1T8I) compared to PST, as indicated by MM-GBSA binding energies of −60.69 kcal/mol and −35.1467 kcal/mol, respectively (Table 1). Among all the tested analogues, 7-Deoxy-*trans*-dihydronarciclasine exhibited the lowest affinity, with a binding energy of −32.6144 kcal/mol. The remaining analogues displayed varying affinities, with 7 of them (**5–10** and **13**) showing superior binding energies (ranging from −63.14 kcal/mol to −85.10 kcal/mol) in comparison to CPT (Table 1). The current study revealed that altering the structure of PST could significantly impact its computationally assessed affinity to Topo I. In general, nitrogenated analogues exhibited higher affinity compared to both PST and the Topo I inhibitor CPT. Moreover, the ionisation of amine functionalities, coupled with the stereochemistry of the successive chiral centres and the number of oxygen functionalities (Supplementary Table S1), emerged as pivotal factors influencing the analogue’s affinity for the enzyme. The 7-hydroxyl group in PST appeared to have a negative impact on its affinity to Topo I, as evidenced by the enhanced affinity of 7-Deoxypancratistatin compared to PST. The binding energies were determined to be −41.33 kcal/mol and −35.15 kcal/mol, respectively. The adverse effect of the 7-hydroxyl group was not observed when carbon numbers 1 and 10b (Figure 1) were sp^2^ hybridised, as seen in the case of Narciclasine and its 7-deoxy analogue, Lycoricidine. Despite the presence of the 7-hydroxyl group, the binding energies for Narciclasine and Lycoricidine were comparable, at −42.56 kcal/mol and −41.26 kcal/mol, respectively. This similarity in binding energies suggests that the negative impact of the 7-hydroxyl group is not significant when these positions (carbons 1 and 10b) are trigonal planar. Furthermore, the oxygen functionality at position 1 was found to have a detrimental effect on PST affinity to Topo I. This was highlighted by the higher affinity of *trans*-Dihydronarciclasine, which lacks such a functionality, as evidenced by the resulting improved binding energy of −44.76 kcal/mol compared to that of PST. A planar geometry at positions 1 and 10b resulted in increased affinity for Topo I, as demonstrated by Narciclasine exhibiting a 1.2-fold decrease in binding energy compared to PST, which possesses a *trans* junction. Having established that the 1-hydroxyl group is not necessary for binding affinity and by comparing the binding energies of Lycoricidine and 7-Deoxy-*trans*-dihydronarciclasine (−41.26 kcal/mol and −32.61 kcal/mol, respectively), one can infer that a tetrahedral geometry at position 10b could have a detrimental impact on the binding affinity to Topo I. However, the presence of the 7-hydroxyl group could offset the adverse impact of such a geometry, as demonstrated by the 1.4-fold increase in the affinity of *trans*-Dihydronarciclasine compared to that of 7-Deoxy-*trans*-dihydronarciclasine. The binding affinities reported here suggest that Narciclasine and Lycoricidine could serve as potential alternative inhibitors for Topo I.

The conversion of the PST 1-hydroxyl group into a benzoyl ester led to increased affinity towards Topo I (PDB: 1T8I). In this scenario, compound **1** (Figure 5), acting as a benzoyl analogue, exhibited a binding energy of −41.28 kcal/mol towards Topo I, surpassing PST’ binding energy by 1.2-fold (Table 1). The solubility of Narciclasine and its derivatives, such as PST, in aqueous solvents or pharmaceutically acceptable media, is often limited. Therefore, Marion et al. incorporated a salifiable nitrogen group at position 1 to improve the aqueous solubility without compromising the cytotoxic activity [27,28]. The resultant analogues can be administered via various routes, including oral, sublingual, parenteral, subcutaneous, intramuscular, intravenous, transdermal, local, or rectal administration, but in reality, preference is given to intravenous or oral routes. The results of the present study indicate that nitrogenated analogues of PST exhibited greater affinity compared to both Narciclasine and PST. Thus, the replacement of the 1-hydroxyl group with nitrogen functionality further increased the affinity of the resulting analogues towards Topo I. For example, compounds **2**, **3,** and **4** (Figure 5) exhibited stronger binding to the enzyme compared to PST, demonstrated by their calculated binding energies of −44.98 kcal/mol, −56.97 kcal/mol, −44.43 kcal/mol, and −35.15 kcal/mol, respectively (Table 1). It is noteworthy that Marion et al. examined the cytotoxic effects of compound **4** on human cell lines, including A549 (lung) and HCT116 (colon), using Narciclasine as a reference standard [27,28]. Compound **4** exhibited enhanced activity against these cell lines, with IC_50_ values of 8.7 nM and 4.7 nM, respectively, compared to 49 nM and 22 nM, respectively, for Narciclasine [27,28]. Given that Narciclasine and **4** displayed comparable binding affinity to Topo I (−42.55 kcal/mole and −44.43 kcal/mol, respectively), it suggests that the reported cytotoxicity of Narciclasine was likely not mediated through a mechanism involving Topo I. Incorporating amino functionality into the benzamido moiety of compound **4**, as seen in compound **7b** (Supplementary Table S1), enhanced its affinity, with a calculated binding energy of −56.35 kcal/mol (Table 1). The binding affinity of **7b** was influenced by the ionisation state of the basic nitrogen, as evidenced by its ammonium counterpart, **7a** (Supplementary Table S1), which bound the enzyme more strongly with a binding energy of −63.14 kcal/mol. Similarly, **6a** and **6b** (Supplementary Table S1) exhibited varying binding affinities. The uncharged amine **6b** demonstrated a binding energy of only −39.52 kcal/mol, in contrast to −64.97 kcal/mol for the ammonium analogue **6a** (Table 1). This difference could be partly attributed to the absence or presence of interactions with ASP 533, a residue known to enhance the affinity of Topo I to the inhibitor [18]. It was observed that ionised analogues **6a** and **7a** formed water molecule-mediated hydrogen bonds, referred to as water bridges, with ASP 533, while such interactions were not observed for the uncharged analogues **6b** and **7b** (Table 1). Compound **5** (Figure 5) exhibited a further enhancement in binding energy, which was −71.34 kcal/mol (Table 1), underscoring the significance of the ionisation state, the size, and the position of the aminoalkyl moiety in its binding affinity to Topo I. The pivotal role of the ammonium functionality and the size of the alkyl group is clearly evident in the results derived from MM-GBSA calculations on the complexes of **8**, **9** and **10** (Figure 5 and Table 1) with Topo I. All of these analogues are derivatives of PST, characterised by a polyamine structure featuring a linear hydrocarbon chain consisting of 15 carbons. In the hydrocarbon chain, three or four of the carbons are substituted with nitrogen atoms, resulting in primary, secondary, or tertiary amine groups. Based on the count, ionisation status, and the type of amines in existence, calculated binding affinities varied from −59.68 kcal/mol to −85.08 kcal/mol (Table 1 and Supplementary Table S1). It seems that an ideal structural feature for enhanced binding affinity involves a hydrocarbon chain consisting of 15 carbons, with three of them substituted by nitrogen atoms, resulting in two secondary and one primary amines, all of which are ionised. In this context, compound **9b** displayed the most negative binding energy (−85.08 kcal/mol), signifying 2.4 times higher affinity than PST (−35.15 kcal/mol) and 1.4 times higher affinity than the Topo I inhibitor CPT (−60.69 kcal/mol). Despite having a lower affinity for Topo I compared to **8a** (−76.35 kcal/mol), the 4-methyl analogue, **13a**, showed a higher affinity (−68.96 kcal/mol) than both PST (−35.15 kcal/mol) and the co-crystallized inhibitor CPT (−60.69 kcal/mol). However, the ionisation status seemed to influence the binding strength with the enzyme’s active site. To illustrate, **13b** (−76.70 kcal/mol), a 4-methyl analogue with one amine in its free base form, exhibited a similar binding affinity to **8a** (Table 1 and Supplementary Table S1). Once again, ionisation emerged as a critical factor influencing binding affinity to Topo I, as demonstrated by the higher affinity observed in the 4-methyl analogue of PST, **12a** (−56.32 kcal/mol), in comparison to its free base counterpart, **12b** (−25.41 kcal/mol). While **12a** showed lower affinity towards Topo I in comparison to **13a**, indicating the significance of the polyamine, it demonstrated superior affinity compared to **16a** (−49.82 kcal/mol) (Table 1 and Supplementary Table S1). This suggests that adjustments to the PST structure for enhanced activity should be approached with caution. Substituting the 4-hydroxy group with a methyl group should be done alongside a reduction in the number of nitrogen and carbon atoms in the polyamine side chain, with careful consideration of the ionisation state. Comparing the binding energies of **5**, **6a**, **7a**, **14a**, **16a** and **18a** (−71.34 kcal/mol, −64.97 kcal/mol, −63.14 kcal/mol, −52.49 kcal/mol, −49.82 kcal/mol, and −48.42, respectively), it is evident that a benzamido-bearing amine functionality along a 2-carbon tether at the *para* position is favoured. Additionally, incorporating the amine functionality as part of a heterocycle diminishes binding affinity. The incorporation of an electron-withdrawing group at the *meta* position of the benzene ring in the benzamido functionality led to enhanced affinity compared to compound **4** (−44.43 kcal/mol), as evidenced by compounds **19** and **20** (−47.15 kcal/mol and −53.38 kcal/mol, respectively) (Figure 5 and Table 1). However, the (alkylamino)methyl group continued to demonstrate superior affinity, as observed in **6a** (−64.97 kcal/mol) and **7a** (−63.14 kcal/mol). An exception to this trend was seen in the binding energy displayed by **18a** (−48.42 kcal/mol), which was comparable to that of **19**. Notably, **18** differs from **6** and **7** in the type of amine and the size of the alkyl group attached to the nitrogen. Unfortunately, the available data is insufficient to determine which structural feature is responsible for the relatively reduced affinity of **18a** to Topo I compared to **6a** and **7a** (Table 1 and Supplementary Table S1).

### 2.4. ADMET Studies

Unsatisfactory absorption, distribution, metabolism, excretion, and toxicity (ADMET) properties are responsible for > 40% of promising drug candidates’ failure during the drug discovery process [44]. Promising molecules with optimal drug-like features and pharmacokinetic properties are more likely to have efficient therapeutic efficacy. As such, compounds having adverse pharmacokinetic properties may be removed from consideration as drug candidates despite high potency. Alternatively, their ADMET properties might be adjusted to enhance their chances of reaching clinical trials [45]. Therefore, carrying out ADMET screening early in drug discovery helps eliminate candidates with poor physicochemical and ADMET properties, allowing the drug discovery research to focus on fewer, more promising molecules [46]. On these grounds, the ADMET properties of the top-ranked compounds, the notable Topo 1 inhibitor CPT and the natural *Amaryllidaceae* alkaloids, were assessed based on their chemical structures using the Qikprop module of Schrödinger suite (Schrödinger Release 2023-2: Maestro, Schrödinger, LLC, New York, NY, USA, 2023.). There are > 50 ADMET parameters from this module, each with specific accepted value ranges. This module predicts the ADMET properties of a drug candidate by generating relevant physical descriptors [47]. As can be seen in Supplementary Table S2, most of the top-ranked compounds had ADME-compliance scores (#stars) below 5, indicating that their property descriptors fell within the acceptable range for approved drugs. Compounds **8–10** and **13**, featuring a polyamine structure with a linear hydrocarbon chain, had #stars > 8, suggesting they poorly align with the properties needed for drug approval and were thus excluded from further consideration. In other words, they were not subjected to molecular dynamics simulations. The investigated compounds did not violate both the rule of five (Ro5) and the rule of 3 (Ro3), and the likely metabolic reactions were within the permissible range [48]. A drug candidate’s bioavailability is determined by its absorption and the presystemic first-pass effect. Absorption, in turn, is influenced by multiple factors, such as the compound’s solubility and permeability, as well as its interactions with the metabolising enzymes and transporters in the GI tract. In this context, multiple parameters were employed to evaluate the oral absorption, including the predicted aqueous solubility, log*S*wat, the conformation-independent predicted aqueous solubility, CI log*S*wat, the predicted qualitative human oral absorption, the predicted % human oral absorption (HOA) (on 0 to 100% scale), and compliance to Ro3 [45]. The predicted HOA of compounds **8–10** and **13** showed 0%, while the others displayed weak to medium absorption rates (2.74% to 77.12%). The reference ligand CPT and PST are predicted to be absorbed at 84% and 29%, respectively. PST and its analogues showed less potential for cardiotoxic effects, reflected by their predicted low IC_50_ values for blocking HERG K^+^ channels. With the exception of the PST nitrogenated derivative **3**, the predicted IC_50_ values for blocking HERG K^+^ channels for all the investigated compounds, including CPT, were <−5, indicating an increased risk of cardiac arrhythmias and potential cardiotoxicity. Compound **3** contains a urea linker, while compound **4** incorporates an amide linker. This could explain the greater cardiotoxic effect of compound **4** compared to compound **3**, as studies have shown that urea is often used to fine-tune crucial drug-like properties [49]. Thus, a urea linker can be used instead of an amide linker to improve the safety profile of the top-ranked PST nitrogenated derivatives identified in this study. Once inside the human body, the drug molecules are either bound to plasma proteins in the bloodstream or are unbound (free) and able to diffuse in the aqueous fluids of the blood and tissues. In most therapeutically useful drugs, only the free fraction can interact with the target to elicit pharmacological effects [50]. In this study, we computed the binding affinity of the investigated compounds to human serum albumin using the QPlogKhsa descriptor. All the investigated derivatives, as well as the reference ligand CPT, showed less potential to bind with human serum albumin (QPlogKhsa < 0). This suggests they can circulate freely in the bloodstream at relatively high concentrations sufficiently enough to exert their therapeutic effects. In order to estimate the ability of the top-ranked compounds to cross the blood-brain barrier (BBB), we used the descriptor QPlogBB (Predicted brain/blood partition coefficient). Based on the results in Supplementary Table S2, all top-ranked compounds showed limited potential to cross the BBB (QPlogBB values < 1), indicating they are CNS-inactive molecules. Moreover, the octanol/water partition coefficient (QPlogPo/w), a parameter used to predict a compound’s lipophilicity, indicated that the investigated PST derivatives were too polar (QPlogPo/w < 1.5). It is known that highly polar molecules do not cross the blood-brain barrier (BBB) [51]. Results of QPlogPo/w calculations indicated that modifying PST (QPlogPo/w = −1.51) improved the lipophilicity of its derivatives **1–20** (QPlogPo/w = −1.19 to 1.19). However, they remain too polar to penetrate the CNS. Furthermore, the estimated CNS activity of the PST and its nitrogenated derivatives, computed on a −2 (inactive) to +2 (active) scale, showed that all these derivatives could be inactive in the CNS (predicted CNS activity = −2). Apparent MDCK cell permeability (QPPMDCK) is frequently used as an additional parameter to estimate a molecule’s ability to penetrate the CNS [45]. With the exception of compound **19**, the QPPMDCK values calculated for the top-ranked compounds fell outside the recommended range of 25 to 500 nm s−1 for 95% of known drugs. This further supports their low potential to cross the BBB. To determine if PST nitrogenated derivatives **1–20** can efficiently reach their target site after entering the bloodstream, the expected number of metabolic reactions was predicted using the parameter (#metab). The predicted number of possible metabolic reactions for these derivatives was within the specified limit (1–8). However, compounds **8–10, 17**, and **20** were predicted to undergo several metabolic reactions, ranging from 9 to 11, before reaching their target. The consequences are: (i) more frequent dosing required, (ii) reduced efficacy due to limited concentration at the target site, and (iii) increased potential of drug-drug interactions [52]. Thus, addressing metabolic instability can involve structural modification of metabolic soft spots, optimising the formulation, or altering the metabolic route [53]. In brief, most of the top-ranked compounds, except for **8–10** and **13**, demonstrated promising drug-likeness and pharmacokinetic profiles. Therefore, they could serve as a valuable foundation for further research.

Topoisomerase enzymes are involved in various essential physiological functions, particularly in tissues with rapidly dividing cells [17,18,19,20,21,22,23,24,25,26]. As a result, off-target effects could be a potential drawback for Topo I inhibitors. Computational models are limited in predicting off-target effects because they cannot account for all biomolecular interactions in living cells. Thus, there is always a limitation of uncertainty regarding the safety of the compounds being investigated. It is recommended that future studies should evaluate the selectivity of the identified PST analogues for Topo I, specifically targeting cancer cells, through experimental approaches.

### 2.5. Molecular Dynamics Simulations

Ligand behaviour undergoes continuous changes in vivo, while molecular docking offers only a static view of ligand-protein interaction. Hence, to bridge this gap, molecular dynamics (MD) simulations should be performed to investigate the dynamic characteristics of the inhibitory mechanism and the stability of the ligand-protein complex [54]. Various characteristics are computed to evaluate the stability of the entire complex with and without the ligand [55]. For instance, the time-dependent root mean square deviations (RMSD) of the backbone atoms of the complex are derived from the X-ray crystal structure of the protein throughout the simulation period to gauge complex stability. Additionally, the root mean square fluctuation (RMSF) serves as a valuable metric in assessing the structural flexibility of the protein’s backbone atoms, both in its complexed and Apo forms [36]. In the present study, docked complexes involving the Topo I enzyme (PDB: 1T8I) with CPT, PST and PST analogues (**5**, **6a** and **7a**), which exhibited superior binding affinities compared to CPT and acceptable ADMET profiles were chosen for MD simulations. These complexes were subjected to a 100 ns simulation aiming to provide a more realistic representation of the interaction patterns between all ligands and 1T8I. The rigid examination of the 1T8I crystal structure was executed through Glide protein-ligand docking computations [56]. Following the binding of all ligands, the temporal structural changes in 1T8I were analysed in terms of RMSD, RMSF, hydrogen bonding and hydrophobic interactions. The results are displayed as the maximum, minimum and average values of each trajectory analysis parameter (Table 2).

**Figure 6:**
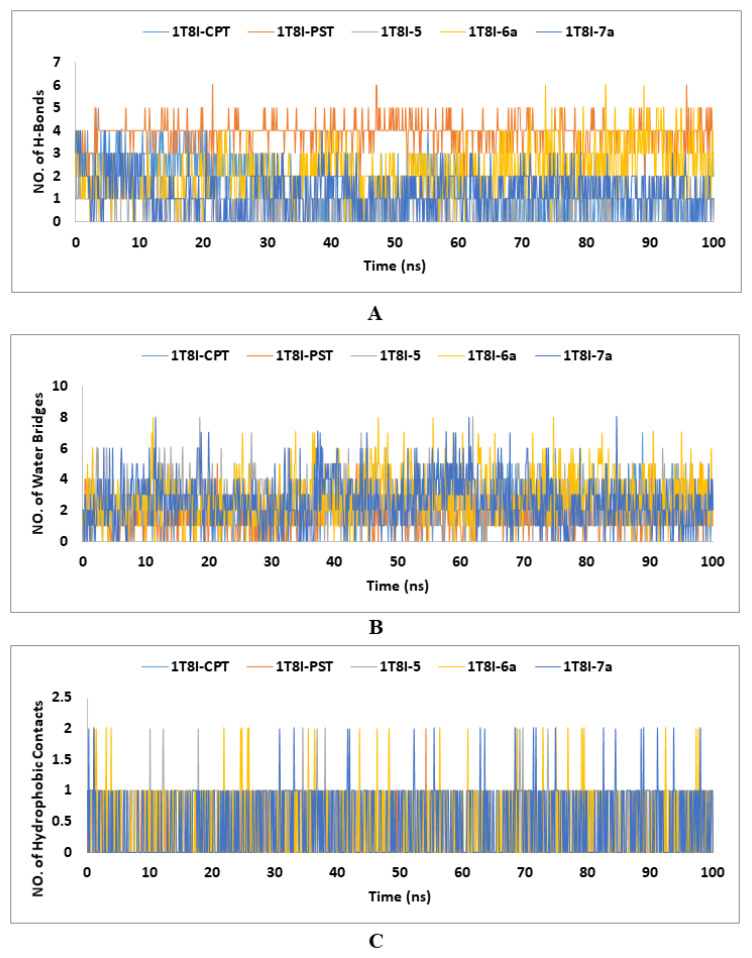
The number of (**A**) H-Bonds, (**B**) water bridges, and (**C**) hydrophobic contacts established during the entire MD simulations run of the CPT, PST and the top-ranked PST analogues (**5**, **6a** and **7a**) with Topo I (PDB: 1T8I).

In the context of ligand-protein complexes in MD simulations, hydrogen bonds are essential for the specificity and strength of the interactions between the ligand and the protein. They contribute to the stability of the complex by forming key interactions that anchor the ligand in the binding site. Hydrogen bonds help define the binding affinity and orientation of the ligand, influencing the overall dynamics and conformational changes within the protein. Accurate modelling of these hydrogen bonds is crucial for understanding the binding mechanisms, predicting binding affinities, and designing effective ligands in drug discovery and other applications [57]. In the investigation of Topo I’s (PDB ID: 1T8I) interactions with various ligands (CPT, PST, **5**, **6a** and **7a**) using MD simulations over 100 ns, the analysis of hydrogen bonds revealed significant insights. PST and analogue **5** formed the highest number of maximum hydrogen bonds (six), suggesting strong and stable interactions, while CPT, the co-crystallized ligand, formed up to five hydrogen bonds, indicating reliable but slightly less extensive interactions. Analogues **6a** and **7a** each formed a maximum of four hydrogen bonds, indicating potentially weaker binding. The minimum hydrogen bond counts showed that only PST maintained at least one hydrogen bond consistently, while the others had moments with no hydrogen bonds, suggesting intermittent interaction stability. The average number of hydrogen bonds for CPT, PST, **5**, **6a** and **7a** were 1.55, 3.87, 2.22, 1.24, and 0.79, respectively. Overall, PST exhibits the strongest binding affinity, followed by **5**, with CPT showing moderate interaction stability and **6a** and **7a** demonstrating relatively weaker interactions (Figure 6A).

Water bridges, or water-mediated hydrogen bonds, are essential in stabilising and dynamically modulating ligand-protein complexes in MD simulations. These water molecules facilitate indirect interactions between the ligand and protein, enhancing binding affinity and specificity by forming adaptable hydrogen bonds that accommodate structural fluctuations. They also influence binding kinetics and contribute significantly to the binding free energy, affecting the overall stability and affinity of the complex [58]. Analysing water bridges in MD simulations reveals their crucial role in maintaining the integrity and functionality of ligand-protein interactions. In the 100 ns MD simulations of the 1T8I-ligand complexes, water bridge contacts ranged from zero to eight, with the co-crystallized ligand CPT having seven contacts and other ligands (PST, **5**, **6a** and **7a**) showing up to eight. Despite this variability, the average number of water bridges was relatively low for all ligands, ranging from 1.59 to 2.82, indicating that water-mediated interactions are present but not predominant. This suggests that while water bridges play a role in stabilising these complexes, their frequency and impact can vary significantly, depending on the specific ligand and its interactions within the protein environment (Figure 6B).

The hydrophobic contact data from the 1T8I-ligand MD simulations reveal that the co-crystallized ligand CPT does not engage in hydrophobic interactions, while other ligands (PST, **5**, **6a**, and **7a**) show some level of hydrophobic contact, with averages ranging from 0.07 to 0.53. This suggests that although hydrophobic interactions are not predominant, they do contribute to the binding characteristics of PST, **5**, **6a,** and **7a**, potentially enhancing their stability and interaction with the protein (Figure 6C).

**Figure 7:**
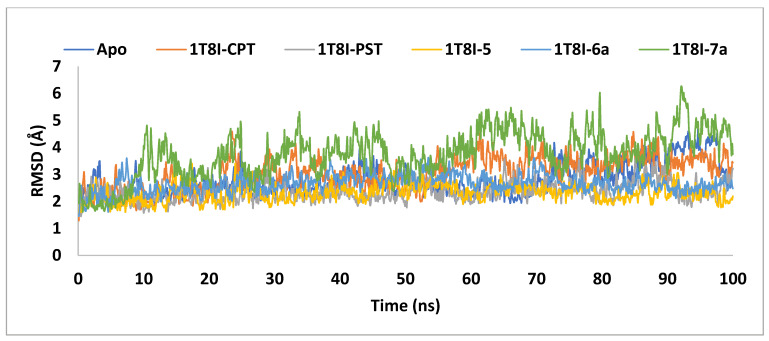
The RMSD analysis of Topo I (PDB: 1T8I) apoprotein and its complexes with CPT, PST, **5**, **6a** and **7a** monitored during the MD simulations trajectories.

In order to clarify the conformational stability of the ligand-1T8I complexes, the time evolution of changes in all atoms’ Cα-RMSD was examined, as depicted in Figure 7. The degree of RMSD variation inversely correlates with a complex’s stability, where lower variation indicates stronger stability [36].

The graph demonstrates that the backbone stability of 1T8I, when complexed with the co-crystal ligand CPT, PST, **5,** or **6a,** remained consistently stable throughout the simulation. In contrast, the **7a** bound 1T8I complex did not achieve satisfactory equilibrium throughout the entire simulation, where it fluctuated between 6.26Å and 1.61Å, with an average value of 3.76Å. The 1T8I-**5** complex exhibited the least disparity between the maximum and minimum RMSD values (1.8Å) and the lowest average (2.31Å), indicating that ligand **5** maintained its binding mode within the protein binding site through preserved interactions [38] (Table 2).

PST exhibited comparatively greater stability in the ligand-protein complex than CPT when binding to 1T8I. The 1T8I-PST complex showed fluctuations between 3.59Å and 1.46Å, averaging 2.36Å. In contrast, the CPT-bound 1T8I complex fluctuated with an average of 3.04Å, with maximum and minimum values of 4.59Å and 1.29Å, respectively (Table 2). Understanding that inhibitors with strong binding energies generally demonstrate potent activity, it is notable that the findings of the current study diverge from this expectation. Specifically, PST exhibited lower affinity but displayed better ligand-protein complex RMSD values compared to CPT despite CPT being recognised as a more potent Topo I inhibitor [16]. This discrepancy can be explained by considering that, during simulation, PST failed to interact with key residues crucial for enzyme activity, such as ARG 364, ASP 533, and ASN 722 (Supplementary Figure S1). In contrast, CPT formed direct or water-mediated hydrogen bonds with these residues for up to 66% of the simulation time (Supplementary Figure S2).

Replacing the PST 1-hydroxyl group with an alkylamino functionalised benzamido moiety allowed for the generation of ionised forms in the resulting analogues. This facilitated interactions with ASP 533 through water bridges, thereby enhancing affinity to the 1T8I binding site. This effect was observed in compounds **5**, **6a** and **7a**, with MM-GBSA binding energies measured at −71.34 kcal/mol, −64.97 kcal/mol and −63.14 kcal/mol, respectively (Table 1). However, the stability of the protein-ligand complex seemed to depend on the position and type of aminoalkyl group, along with the number of carbon atoms between the benzene and the nitrogen atom. Introducing an *n*-butyl substituent on the nitrogen demonstrated superior performance in terms of complex stability compared to a cyclopropyl substituent. In this scenario, while **6a** showcased good stability with an average RMSD value of 2.67Å, analogue **7a** exhibited unsatisfactory stability with an average RMSD value of 3.76Å (Table 2). Moreover, attaching the *n*-butyl amine to the benzamido ring via two carbon atoms at the *para* position proved advantageous not only for enhancing binding affinity but also for stabilising the ligand-protein complex. In this context, the 1T8I complexed with **6a** displayed a binding energy of −64.97 kcal/mol with an average RMSD value of 2.67Å, whereas the 1T8I-**5** complex presented a binding energy of −71.34 kcal/mol with an average RMSD value of 2.31Å.

Throughout the simulation period, several interactions occurred between **5** and 1T8I. Notably, a hydrogen bond formed with the key residue ASN 352, maintaining stability for approximately 44% of the simulation duration. Furthermore, two hydrogen bonds mediated by water molecules were observed with the crucial residue ASN 722, persisting for 48% and 56% of the simulation time, respectively. This finding aligns well with previous studies reported in the literature, which also highlight the significance of water-mediated hydrogen bonds between inhibitors and ASN 722 [22]. It is worth mentioning that mutations in ASN 722 have been linked to enzyme resistance against certain inhibitors [18,22,59]. A hydrophobic interaction of **5** with PRO 431, present for 52% of the simulation duration, contributed significantly to the stabilisation, in terms of RMSD, of the 1T8I-**5** complex (Supplementary Figure S3).

Similar interactions were observed for **6a**, where it formed a hydrogen bond with ASN 722 and a hydrophobic interaction with PRO 431, persisting for 61% and 31% of the simulation duration, respectively. Although the hydrogen bond between **6a** and ASN 352 lasted for 17% of the time, its interaction with ALA 351 via water bridge remained intact for 32% of the simulation. In contrast to **5**, analogue **6a** engaged in a hydrogen bond with LYS 436 for 59% of the simulation duration (Supplementary Figure S4).

During the simulation, CPT established hydrogen bonds with the crucial residues ARG 364 and ASP 533, persisting for 66% and 33% of the simulation time, respectively. Furthermore, two water bridges were noted between CPT and ARG 488, as well as ASP 533, spanning 69% and 46% of the simulation time, respectively (Supplementary Figure S2).

The residue ASP 533, crucial for enzyme sensitivity to CPT, has been associated with enzyme resistance to inhibitors following mutation [18,22]. Additionally, genetic and structural data strongly suggest that mutation removal of ARG 364 to HIS reduces inhibitor affinity for the Topo I—DNA complex by eliminating an essential contact [18]. In contrast, PST did not exhibit any interactions with ARG 364 or ASP 533 throughout the simulation. This observation could explain the comparatively weak Topo I inhibitory activity reported by Ding et al. [16] in comparison to CPT. It is notable that analogues **5** and **6a** engaged with ARG 364 and ASP 533 throughout the simulation via both direct hydrogen bonds and water-mediated hydrogen bonds. Additionally, they formed ionic interactions exclusively with ASP 533 (Supplementary Figures S5 and S6).

**Figure 8:**
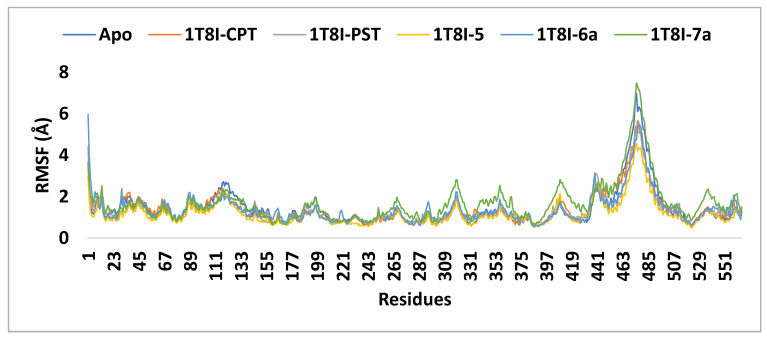
The RMSF analysis of the 1T8I apoprotein and its complexes with CPT, PST, **5**, **6a** and **7a** monitored during the MD simulation trajectories.

The RMSF (root mean square fluctuation) values were computed to assess the structural flexibility of the protein’s backbone atoms in both the complex and the Apo form. The analysis revealed that analogue **5** exhibits better inhibitory potential not only compared to **6a** and **7a** but also surpassing that of CPT and PST. As depicted in Figure 8, the small RMSF values observed for the 1T8I-**5** complex, compared to **6a**, CPT, and PST complexes, indicate stable and robust interactions between 1T8I and analogue **5**. These interactions result in reduced fluctuation of active site residues [18] (i.e., for 1T8I-**5** complex: the RMSF values (Å) of ASN 722, THR 718, ASP 533, LYS 532, ARG 364, GLU 356 and ASN 352 are 0.52, 0.69, 0.64, 0.58, 0.74, 0.99 and 0.77, respectively, while for 1T8I-**6a** are 0.65, 0.80, 1.00, 0.83, 1.35, 1.18, and 1.22 and for 1T8I-PST complex are 0.62, 0.90, 1.03, 0.97, 0.80, 0.75 and 0.84, respectively, for 1T8I-CPT complex are 0.62, 0.75, 0.97, 1.05, 0.87, 1.16 and 1.03, respectively, and for Apo are 0.59, 0.70, 0.65, 0.61, 0.83, 1.39 and 1.40, respectively).

The diminished RMSD and RMSF fluctuations observed for **5** and **6a** when complexed with 1T8I, alongside their persistent interactions with key residues throughout the simulation, suggest their potential as candidates for inhibiting Topo I. Additionally, their lower MM-GBSA binding energy compared to CPT and PST further supports this proposition. It is noteworthy that Marion et al. [27,28] reported the syntheses of compounds **5** and **6a**, among other nitrogenated derivatives of PST. They revealed that introducing a nitrogenated group at position 1 of PST enabled the creation of derivatives with cytotoxic properties similar or superior (up to 10 times) to those of Narciclasine, along with enhanced solubility in aqueous solvents. Indeed, the incorporation of a salifiable nitrogenated group facilitates increased solubility of the compounds without compromising their cytotoxic activity.

### 2.6. Study Limitations and Future Perspective

This study has identified nitrogen-based analogues of PST as promising anticancer candidates targeting Topo I. A thorough in silico approach, including molecular docking, MM-GBSA analysis, and MD simulations, provides valuable insights into the binding interactions and stability of these analogues. The findings reveal key structural features associated with affinity to Topo I and ligand-receptor complex stability, while computational ADMET predictions emphasise the potential of these analogues for further development. Notwithstanding, several limitations must be addressed. The accuracy of in silico models depends on available structural data and simulations, which may not fully capture the complexities of biological systems. Computational models are limited in predicting a compound’s behaviour in living organisms, as they cannot account for all biomolecular interactions, such as off-target effects or metabolic pathways, creating uncertainty regarding the efficacy and safety of the compounds being tested.

To mitigate this, the in silico findings of the current study must be validated through in vitro and in vivo studies. Biological experiments, including synthetic optimisation of compounds **5** and **6** with the same chirality, enzyme inhibition assays, cytotoxicity tests on cancer cell lines, and solubility assessments, are necessary to confirm the efficacy, safety, and practicality of the identified lead compounds. The optimisation of analogues **5** and **6** should adopt a systematic approach that focuses on designing new analogues based on the essential structural features identified. Constructing a pharmacophore model, followed by molecular docking and QSAR modelling, will aid in predicting the effects of structural modifications on the binding affinity and pharmacokinetics of the newly designed analogues. To enhance binding affinity and stability with crucial residues like ASP 533 and ASN 352, modifications should target optimising the ionisation state of the amine group to strengthen electrostatic interactions with key residue ASP 533, specifically by adjusting the pKa for ideal ionisation at physiological pH. Tuning stereochemistry is also key to ensuring the molecule fits well within the active site and forms optimal bonds. Additionally, adjusting the number and position of oxygen functionalities can improve hydrogen bonding with ASN 352. Furthermore, simplification of 5 and 6 structures while keeping key functional features like amine and oxygen groups can make the structure easier to synthesise without losing efficacy. This not only simplifies synthesis but can also enhance drug-like properties, including solubility and stability. Finally, the most promising candidates will then be synthesised and subjected to experimental validation.

## 3. Materials and Methods

The Schrödinger suite includes several embedded software tools: Protein Preparation Wizard [39], LigPrep (Schrödinger Release 2023-2: LigPrep, Schrödinger, LLC, New York, NY, USA, 2023.), Glide [56], Prime [60], Qikprop [61], and Desmond [62]. These tools were accessed through the Maestro graphical interface (Schrödinger Release 2023-2: Maestro, Schrödinger, LLC, New York, NY, USA, 2023).

### 3.1. Molecular XP Docking

The structures of Pancratistatin’s analogues were downloaded from PubChem [63] and the relevant literature. Glide XP docking of the prepared database was then performed. The downloaded analogues, initially in 2D SDF format, were converted to 3D format and optimised using the OPLS4 force field. The LigPrep module of Schrödinger was used to prepare the structures for docking, retaining the original chiralities of the ligands. Epik was employed to generate possible ionisation states at pH 7.00 ± 2 units, producing one low-energy conformer for each ligand. The 3D crystal structures of the Topo I enzyme (PDB ID: 1T8I) and its respective co-crystal ligand were retrieved from the Protein Data Bank (www.rcsb.org, accessed on 10 March 2023) [18]. The protein structure was prepared using the multistep Protein Preparation Wizard (PrepWizard) from Maestro Task. All water molecules were removed, and the co-crystal ligand was retained in the active site. Optimisation and energy minimisation were carried out using the OPLS4 force field. A 3D cubic grid box was created around the co-crystal ligand using the receptor grid generation tool within the Maestro suite. Subsequently, the prepared ligands were screened against the refined target protein using the Glide XP module of the Schrödinger suite. One best pose was generated for each input molecule and these molecules were ranked based on their Glide docking score.

### 3.2. MM-GBSA Binding Free Energy Calculations

The Prime module, integrated with Schrödinger, was used to estimate the binding free energy of the receptors and docked ligands. The Pose Viewer Files (PVF) generated post-docking were used as input files to calculate the binding free energy for each compound. The VSGB 2.0 solvation model and OPLS4 force field were employed to compute the free energy descriptors, following a previously reported protocol [38]. The MM-GBSA ∆G binding energy score was utilised to rank the ligands.

### 3.3. ADMET Studies

In this study, the computational tool QikProp was used to determine the ADMET profiles and drug-likeness features of all investigated ligands. All calculated parameters were evaluated for compliance with their respective standard ranges.

### 3.4. Molecular Dynamics Simulations

A molecular dynamics (MD) simulation study was conducted using the Desmond MD simulation package for the best ligand-target complexes, including the co-crystallized ligand, PST, **5**, **6a** and **7a** from the docking experiments. Initially, the SPC solvation model was employed and the system was placed within an orthorhombic water box with dimensions of 10 Å × 10 Å × 10 Å, to ensure complete coverage of each complex with the solvent model. Na^+^ counter ions were added to the systems to balance the net charges and 0.15 M NaCl was added to neutralise the systems. The systems were then minimised and pre-equilibrated using the default relaxation protocol in the Desmond package. The temperature was maintained at 300 K and the atmospheric pressure at 1 bar using the isothermal–isobaric (NPT) ensemble. A 100 ns simulation was carried out, generating 1000 frames of data at 100 ps intervals.

## 4. Conclusions

In conclusion, this study investigated the potential of nitrogenated analogues of PST as new anticancer agents targeting the Topo I enzyme. Employing molecular modelling techniques, it pinpointed promising candidates with superior binding affinities and advantageous interactions with Topo I compared to CPT. Structural analysis unveiled pivotal molecular factors contributing to enhanced potency. In silico predictions indicated favourable drug-like attributes and reduced toxicity. These findings underscore the importance of nitrogenation in augmenting (+)-Pancratistatin anticancer effectiveness and suggest analogues **5** and **6** as lead compounds for further investigations. In this context, validating the in silico findings through comprehensive in vitro and in vivo studies is essential for advancing drug discovery. Systematic optimisation of these analogues, alongside pharmacophore modelling and structural modifications, will improve selectivity, reduce toxicity and enhance solubility. The application of these methodologies will represent significant progress in developing innovative and effective anticancer therapies with improved pharmacological properties.

## Figures and Tables

**Table 1:** Docking scores and MM-GBSA energies of investigated ligands *.

Compound	Docking Score (kcal/mol)	MM-GBSA dG Bind (kcal/mol)
CPT	−8.873	−60.6912
PST	−7.053	−35.1467
7-Deoxypancratistatin	−7.15	−41.3298
Narciclasine	−7.697	−42.5571
Trans-Dihydronarciclasine	−7.096	−44.758
7-Deoxy-trans dihydronarciclasine	−6.222	−32.6144
Lycoricidine	−7.14	−41.2635
Lycorine salt	−6.523	−40.6975
Lycorine	−3.067	−25.1096
1	−7.501	−41.284
2	−9.261	−44.9765
3	−7.26	−56.9672
4	−8.441	−44.4248
5	−10.516	−71.341
6a	−10.802	−64.970
6b	−6.640	−39.520
7a	−10.972	−63.1436
7b	−6.473	−56.3509
8a	−15.789	−76.3536
8b	−12.895	−60.6982
9a	−12.655	−59.6829
9b	−10.619	−85.081
10a	−15.435	−82.5724
10b	−12.501	−67.2082
10c	−11.849	−79.001
10d	−11.675	−73.5333
11	−7.834	−45.5122
12a	−10.357	−56.3187
12b	−4.462	−25.4117
13a	−11.296	−68.9615
13b	−10.163	−76.7022
14a	−9.64	−52.49
14b	−6.915	−37.25
15a	−6.600	−35.89
15b	−6.788	−31.98
15c	−7.452	−31.12
16a	−8.888	−49.82
16b	−6.880	−36.30
17a	−8.693	−46.49
17b	−8.023	−34.60
17c	−6.462	−50.45
17d	−6.344	−18.59
18a	−9.167	−48.42
18b	−5.811	−51.04
19	−7.052	−47.15
20	−8.934	−53.38

* The letters (a, b, c, d) next to compound numbers indicate different ionisation states. For ionised structures, refer to supplementary Table S1.

**Table 2:** A detailed analysis of the RMSD and protein-ligand contacts values, including minimum, maximum, and average for the top-ranked compounds complexes (**5**, **6a** and **7a**) with Topo I (PDB: 1T8I). This also includes comparative data for the CPT and PST complexes, as well as the apoprotein.

	Apo	1T8I-CPT	1T8I-PST	1T8I-5	1T8I-6a	1T8I-7a
PL-RMSD (Å)						
Maximum	4.74	4.59	3.59	3.40	3.61	6.26
Minimum	1.65	1.29	1.46	1.60	1.45	1.61
Average	2.80	3.04	2.36	2.31	2.67	3.76
H-Bond Contact Count						
Maximum	-	5	6	6	4	4
Minimum	-	0	1	0	0	0
Average	-	1.55	3.87	2.22	1.24	0.79
Water Bridge Contact Count						
Maximum	-	7	5	8	8	8
Minimum	-	0	0	0	0	0
Average	-	1.94	1.59	2.82	2.54	2.62
Hydrophobic Contact Count						
Maximum	-	0	2	2	2	2
Minimum	-	0	0	0	0	0
Average	-	0	0.07	0.40	0.46	0.53

## Data Availability

The original contributions presented in the study are included in the article/Supplementary Material, and further inquiries can be directed to the corresponding author/s.

## Supplementary Materials

The following supporting information can be downloaded at: https://www.mdpi.com/article/10.3390/ijms251910779/s1.
