# Supplementary material for: Unveiling the Anticancer Potential: Computational Exploration of Nitrogenated Derivatives of (+)-Pancratistatin as Topoisomerase I Inhibitors
Source: Int J Mol Sci. 2024 Oct 7;25(19):10779. doi: 10.3390/ijms251910779 (PMC11476810; doi:10.3390/ijms251910779)
Supplement: Supplementary file 1 [file ijms-25-10779-s001.zip › ijms-3247107-supplementary.pdf]

Unveiling the Anticancer Potential: Computational Exploration of Nitrogenated Derivatives of (+)-Pancratistatin as Topoisomerase I Inhibitors

Magdi Awadalla Mohamed[1*], Tilal Elsaman[1*], Abozer Y. Elderdery[2], Abdullah Alsrhani[2], Heba Bassiony Ghanem[2], Majed Mowanes Alruwaili[3], Siddiqa M. A. Hamza[4], Salma Elhadi Ibrahim Mekki[5], Hazim Abdullah Alotaibi[6], Jeremy Mills[7]

[1]*Department of Pharmaceutical Chemistry, College of Pharmacy, Jouf University, Sakaka 72388, Saudi Arabia*

[2]*Department of Clinical Laboratory Sciences, College of Applied Medical Sciences, Jouf University, Sakaka 42421, Saudi Arabia*

[3]*Nursing Administration & Education Department, College of Nursing, Jouf University, Sakaka 72388, Saudi Arabia*

[4]*Department of Pathology, College of Medicine, Umm Alqura University, Algunfudah 21912, Saudi Arabia*

[5]*Deparment of Physiology ,College of Medicine, Umm Alqura University, Alqunfudah 21912, Saudi Arabia*

[6]*Department of Medical Oncology, Prince Mohammad Medical City, Aljouf, Saudi Arabia*

[7]*School of Medicine, Pharmacy and Biomedical Sciences, University of Portsmouth, Portsmouth PO1 2DT, UK*

*To whom correspondence should be addressed: M.A.M. (maelhussein@ju.edu.sa); T.E. (telbashir@ju.edu.sa)

Table S1: Chemical structures, molecular interactions, docking scores and MM-GBSA energies of the investigated ligands.

Compound	Pose	Interactions	Docking score (kcal/mol)	MM-GBSA dG Bind (kcal/mol)
**CPT**	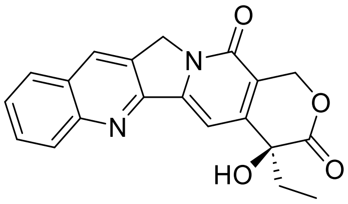	**H-bond:** ASP 533 (1.95Å) **Salt bridge:** None **Pi-Pi stacking:** DT 10 (4.29Å), DC 112 (3.86Å), DA 113 (3.55Å, 3.68Å and 4.05Å) **Pi-cation:** None	−8.873	−60.6912
**PST**	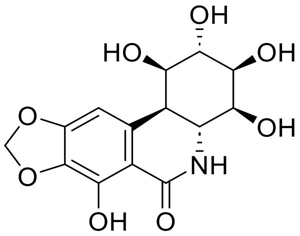	**H-bond:** ASN 352 (2.21Å), TYR 426 (1.85Å and 2.03Å), MET 428 (1.94Å) **Salt bridge:** None **Pi-Pi stacking:** None **Pi-cation:** None	−7.053	−35.1467
**7-Deoxypancratistatin**	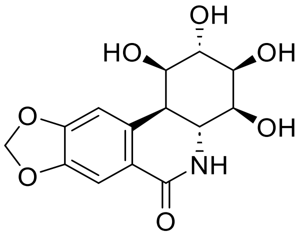	**H-bond:** DT 10 (2.33Å) **Salt bridge:** None **Pi-Pi stacking:** DT 10 (4.12Å and 4.37Å) **Pi-cation:** None	−7.15	−41.3298

**Narciclasin e**	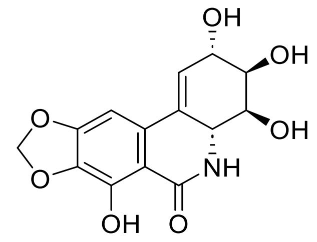	**<u>H-bond:</u>**<br>TYR 426 (2.06Å and 2.08Å), MET 428 (2.08Å), DA 113 (2.19Å)<br>**<u>Salt bridge:</u>**<br>None<br>**<u>Pi-Pi stacking:</u>**<br>None<br>**<u>Pi-cation:</u>**<br>None	−7.697	−42.5571
**Trans-Dihydronar ciclasine**	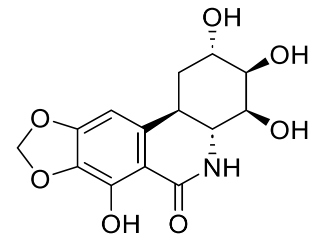	**<u>H-bond:</u>**<br>ASN 722 (1.98Å)<br>**<u>Salt bridge:</u>**<br>None<br>**<u>Pi-Pi stacking:</u>**<br>DT 10 (3.66Å and 3.81Å)<br>**<u>Pi-cation:</u>**<br>None	−7.096	−44.758
**7-Deoxy-trans-dihydronar ciclasine**	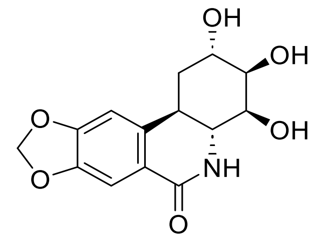	**<u>H-bond:</u>**<br>ARG 364 (2.17Å), DT 10 (1.92Å)<br>**<u>Salt bridge:</u>**<br>None<br>**<u>Pi-Pi stacking:</u>**<br>DT 10 (4.32Å), DA 113 (4.40Å)<br>**<u>Pi-cation:</u>**<br>None	−6.222	−32.6144
**Lycoricidin e**	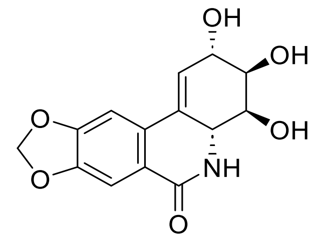	**<u>H-bond:</u>**<br>LYS 436 (2.74Å), TYR 426 (1.87Å and 2.28Å), MET 428 (1.90Å), DA 113 (2.14Å)<br>**<u>Salt bridge:</u>**<br>None<br>**<u>Pi-Pi stacking:</u>**<br>None<br>**<u>Pi-cation:</u>**<br>None	−7.14	−41.2635

**Lycorine salt**	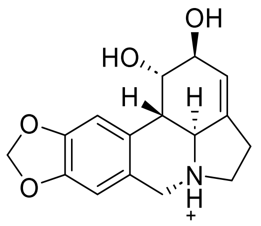	**H-bond:**<br>DT 10 (2.05Å)<br>**Salt bridge:**<br>None<br>**Pi-Pi stacking:**<br>None<br>**Pi-cation:**<br>None	−6.523	−40.6975
**Lycorine**	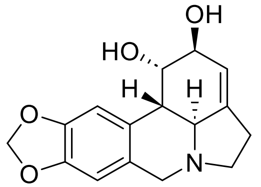	**H-bond:**<br>GLU 356 (1.76Å), ARG 364 (2.27Å)<br>**Salt bridge:**<br>None<br>**Pi-Pi stacking:**<br>DC 112 (3.83Å), DA 113 (3.56Å, 3.57Å and 3.75Å)<br>**Pi-cation:**<br>None	−3.067	−25.1096
**1**	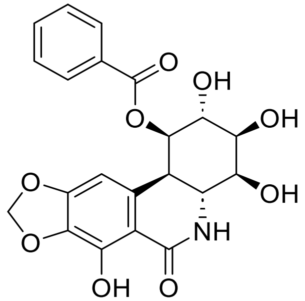	**H-bond:**<br>ARG 364 (2.39Å), ASN 352 (2.37Å), DT 10 (2.01Å and 2.40Å)<br>**Salt bridge:**<br>None<br>**Pi-Pi stacking:**<br>DC 112 (3.91Å)<br>**Pi-cation:**<br>LYS 425 (4.57Å and 5.19Å)	−7.501	−41.284
**2**	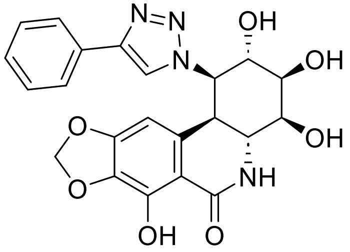	**H-bond:**<br>LYS 425 (1.98Å), TYR 426 (2.31Å), ASN 352 (2.03Å), DA 113 (2.17Å)<br>**Salt bridge:**<br>None<br>**Pi-Pi stacking:**<br>DA 113 (4.17Å)<br>**Pi-cation:**<br>None	−9.261	−44.9765

**3**	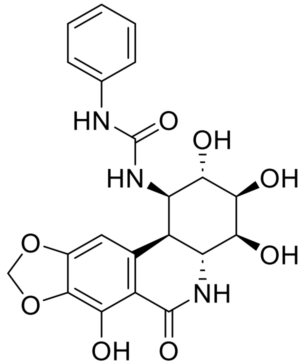	**H-bond:**<br>ASN 352 (2.34Å),<br>**Salt bridge:**<br>None<br>**Pi-Pi stacking:**<br>DT 10 (3.78Å and 3.85Å)<br>**Pi-cation:**<br>None	−7.26	−56.9672
**4**	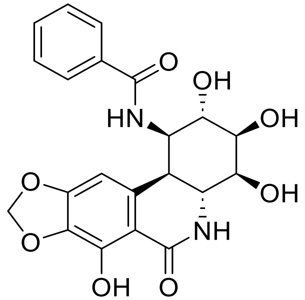	**H-bond:**<br>TYR 426 (2.25Å), ASN 352 (1.98Å), DA 113 (2.16Å and 2.49Å)<br>**Salt bridge:**<br>None<br>**Pi-Pi stacking:**<br>None<br>**Pi-cation:**<br>None	−8.441	−44.4248
**5**	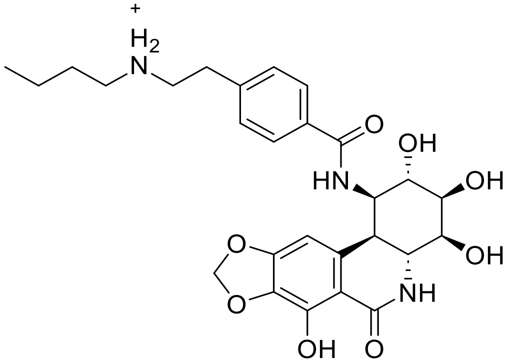	**H-bond:**<br>LYS 436 (1.96Å and 2.16Å), ASP 533 (2.23Å)<br>**Salt bridge:**<br>ASP 533 (3.06Å)<br>**Pi-Pi stacking:**<br>DT 10 (3.66Å)<br>**Pi-cation:**<br>None	−10.516	−71.341

**6a**	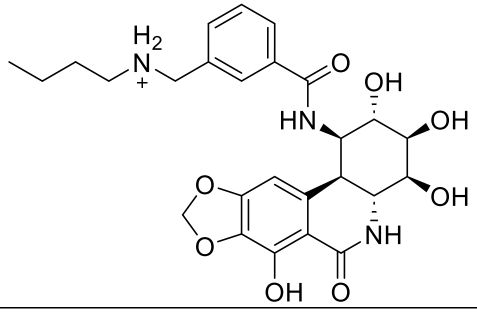	**<u>H-bond:</u>**<br>LYS 436 (2.03Å and 2.23Å)<br>**<u>Salt bridge:</u>**<br>ASP 533 (4.90Å)<br>**<u>Pi-Pi stacking:</u>**<br>DT 10 (3.68Å)<br>**<u>Pi-cation:</u>**<br>None	−10.802	−64.970
**6b**	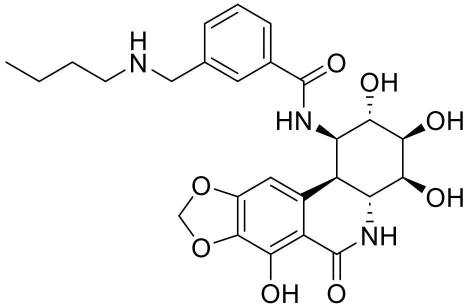	**<u>H-bond:</u>**<br>ASN 352 (2.07Å), LYS 436 (1.85Å), THR 718 (1.81Å)<br>**<u>Salt bridge:</u>**<br>None<br>**<u>Pi-Pi stacking:</u>**<br>DT 10 (3.99Å)<br>**<u>Pi-cation:</u>**<br>None	−6.640	−39.520
**7a**	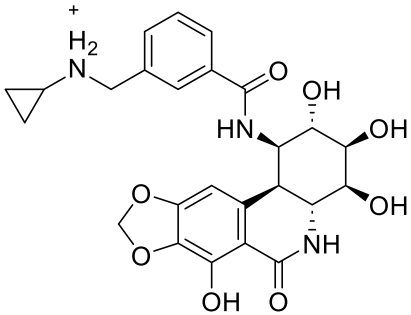	**<u>H-bond:</u>**<br>LYS 436 (2.02Å and 2.30Å), LYS 751 (2.16Å)<br>**<u>Salt bridge:</u>**<br>ASP 533 (4.75Å)<br>**<u>Pi-Pi stacking:</u>**<br>DT 10 (3.69Å)<br>**<u>Pi-cation:</u>**<br>None	-10.972	−63.1436
**7b**	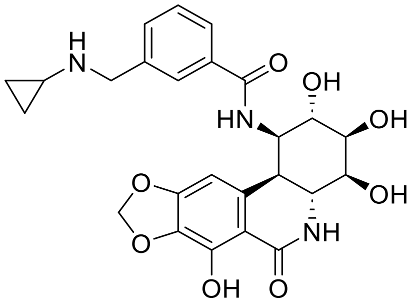	**<u>H-bond:</u>**<br>LYS 436 (2.03Å and 2.25Å), LYS 751 (2.21Å), DT 10 (1.96Å)<br>**<u>Salt bridge:</u>**<br>None<br>**<u>Pi-Pi stacking:</u>**<br>DT 10 (3.67Å)<br>**<u>Pi-cation:</u>**<br>None	−6.473	−56.3509

**8a**	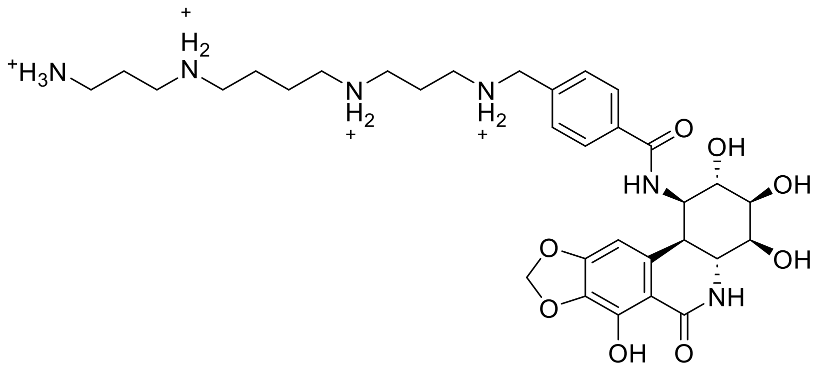	**H-bond:** ASP 533 (2.08Å), TYR 426 (2.40Å), ASN 352 (2.12Å), DA 13 (2.51Å), DA 113 (1.75Å and 1.94Å) **Salt bridge:** ASP 533 (3.01Å) **Pi-Pi stacking:** DT 10 (4.31Å) **Pi-cation:** DT 10 (4.30Å)	−15.789	−76.3536
**8b**	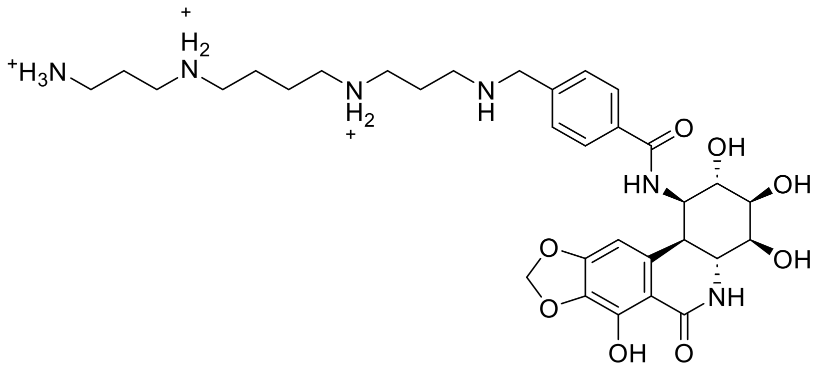	**H-bond:** TYR 426 (1.89Å), LYS 425 (2.19Å), ASN 352 (1.73Å), ASP 533 (1.94Å), DG 12 (1.98Å), DA 13 (1.62Å) **Salt bridge:** ASP 533 (2.81Å) **Pi-Pi stacking:** None **Pi-cation:** None	−12.895	−60.6982
**9a**	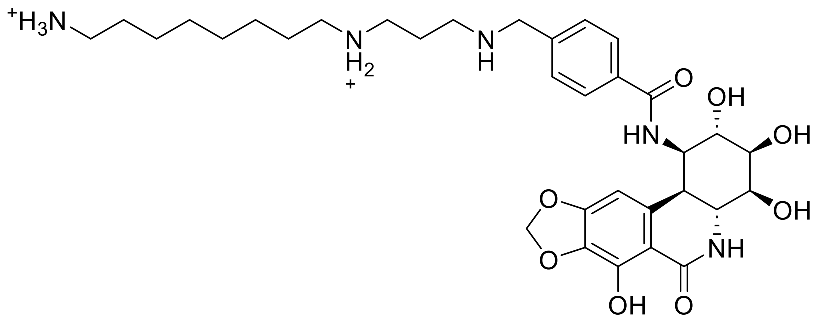	**H-bond:** ASN 352 (1.88Å), TYR 426 (2.15Å), ASP 533 (2.32Å), DA 13 (1.95Å), DA 14 (2.64Å), DA 113 (2.00Å and 2.02Å) **Salt bridge:** ASP 533 (3.23Å) **Pi-Pi stacking:** None **Pi-cation:** None	−12.655	−59.6829

**9b**	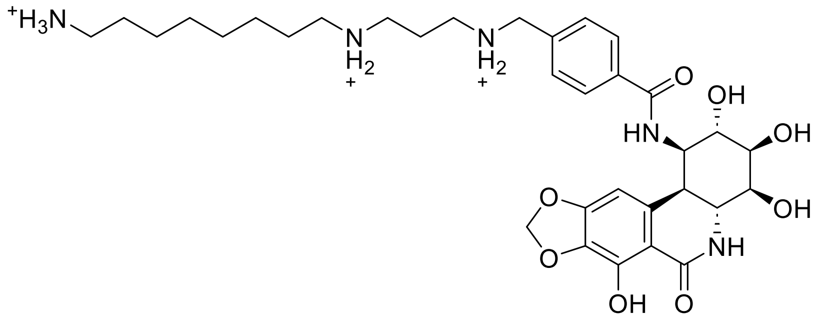	**H-bond:**<br>LYS 436 (2.05Å and 2.17Å), LYS 751 (2.27Å), DG 12 (1.90Å), DC 111 (2.54Å)<br>**Salt bridge:**<br>ASP 533 (4.15Å)<br>**Pi-Pi stacking:**<br>DT 10 (3.65Å)<br>**Pi-cation:**<br>None	−10.619	−85.081
**10a**	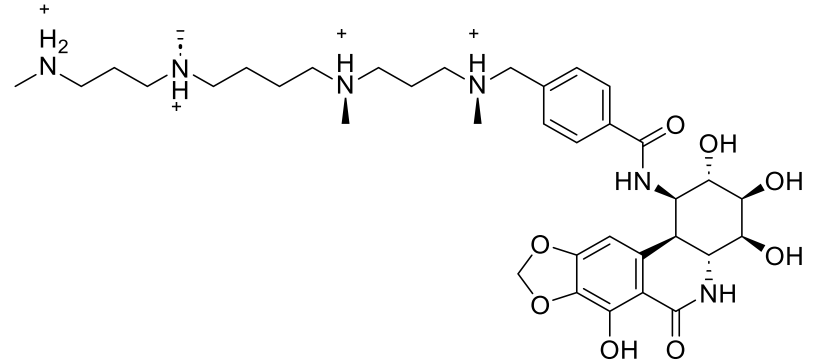	**H-bond:**<br>LYS 436 (2.06Å and 2.13Å), DG 12 (1.84Å), DC 111 (1.87Å and 2.00Å)<br>**Salt bridge:**<br>ASP 533 (4.22Å)<br>**Pi-Pi stacking:**<br>DT 10 (3.64Å)<br>**Pi-cation:**<br>DA 14 (6.59Å)	−15.435	−82.5724
**10b**	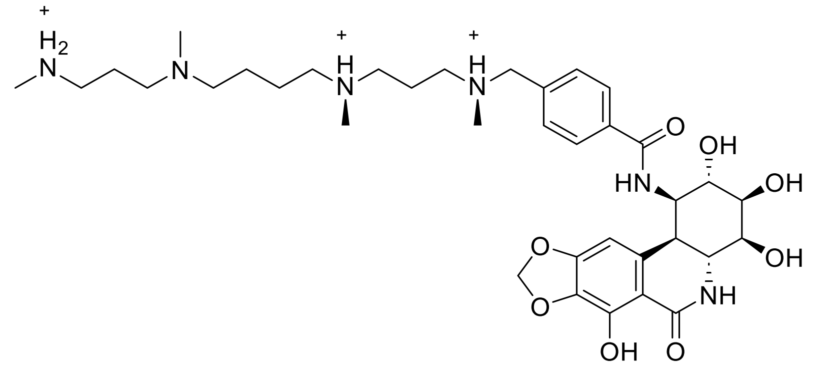	**H-bond:**<br>ASN 352 (2.09 Å and 2.73Å), LYS 425 (2.16Å), TYR 426 (2.33Å), DG 12 (1.82Å), DA 13 (1.87Å), DA 113 (1.89Å and 2.34Å)<br>**Salt bridge:**<br>ASP 533 (3.41Å), DA 13 (4.67Å)<br>**Pi-Pi stacking:**<br>None<br>**Pi-cation:**<br>DT 10 (4.15Å)	−12.501	−67.2082

**10c**	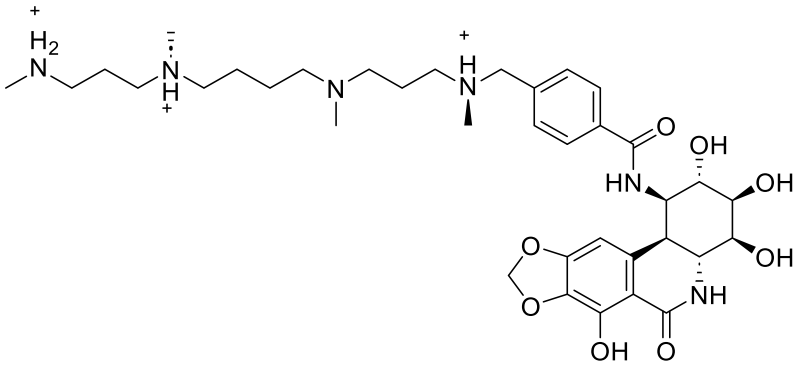	**<u>H-bond:</u>**<br>LYS 436 (2.06Å and 2.24Å), DT 110 (2.19Å), DC 111 (2.05Å)<br>**<u>Salt bridge:</u>**<br>ASP 533 (4.05Å)<br>**<u>Pi-Pi stacking:</u>**<br>DT 10 (3.63Å)<br>**<u>Pi-cation:</u>**<br>DA 15 (6.52Å)	−11.849	−79.001
**10d**	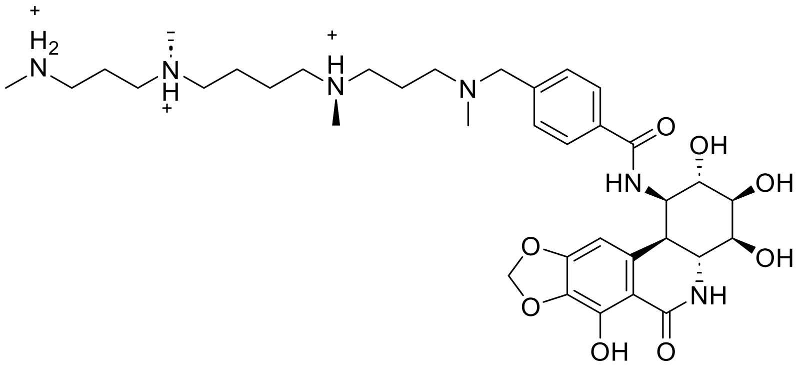	**<u>H-bond:</u>**<br>LYS 436 (2.04Å and 2.18Å), LYS 751 (2.25Å), DG 12 (2.40Å), DT (2.26Å), DC (2.19Å)<br>**<u>Salt bridge:</u>**<br>None<br>**<u>Pi-Pi stacking:</u>**<br>DT 10 (3.65Å)<br>**<u>Pi-cation:</u>**<br>DA 15 (6.52Å)	−11.675	−73.5333
**11**	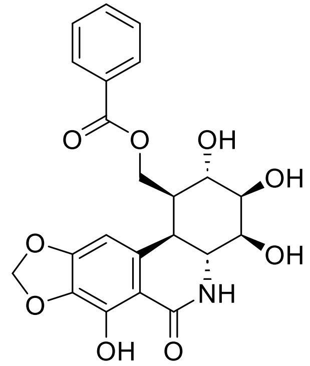	**<u>H-bond:</u>**<br>TYR 426 (1.80Å and 1.92Å), GLU 356 (1.86Å)<br>**<u>Salt bridge:</u>**<br>None<br>**<u>Pi-Pi stacking:</u>**<br>DA 113 (3.71Å)<br>**<u>Pi-cation:</u>**<br>None	−7.834	−45.5122

**12a**	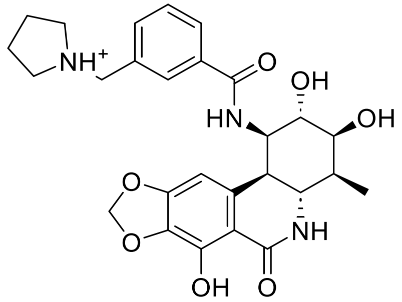	**H-bond:**<br>LYS 436 (1.89Å and 2.51Å), LYS 751 (2.00Å)<br>**Salt bridge:**<br>ASP 533 (4.35Å)<br>**Pi-Pi stacking:**<br>DT 10 (3.66Å)<br>**Pi-cation:**<br>None	−10.357	−56.3187
**12b**	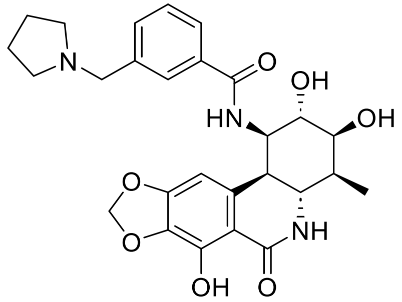	**H-bond:**<br>ASN 352 (2.28Å), LYS 436 (1.86Å)<br>**Salt bridge:**<br>None<br>**Pi-Pi stacking:**<br>DA 113 (3.89Å)<br>**Pi-cation:**<br>None	−4.462	−25.4117
**13a**	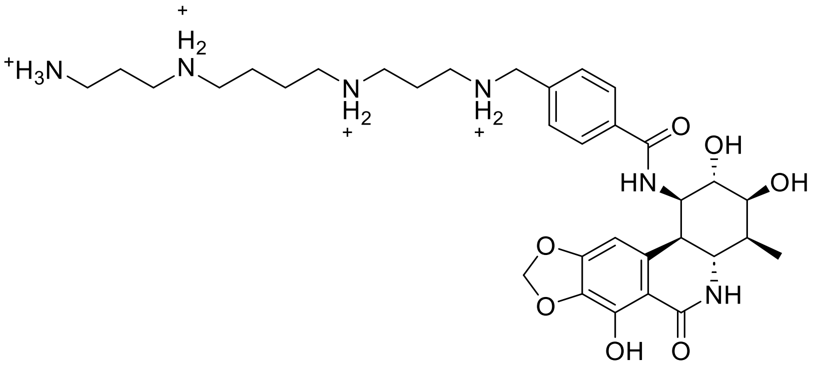	**H-bond:**<br>ASN 352 (2.08Å), LYS 436 (1.86Å), DC 111 (2.14Å)<br>**Salt bridge:**<br>ASP 533 (4.04Å)<br>**Pi-Pi stacking:**<br>DT 10 (3.81Å)<br>**Pi-cation:**<br>DA 14 (6.44Å)	−11.296	−68.9615

**13b**	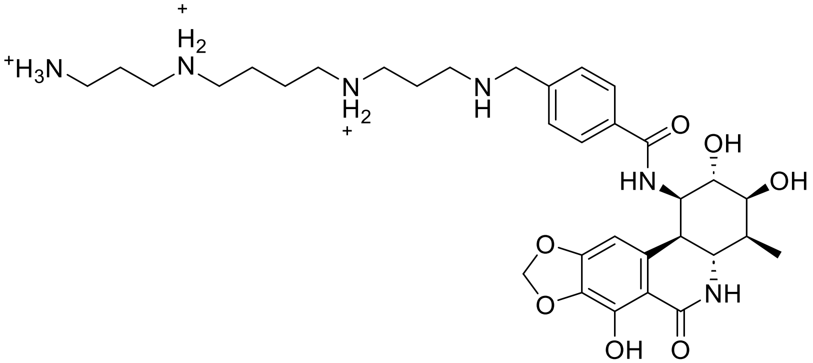	**H-bond:**<br>LYS 436 (1.87Å and 2.25Å), LYS 751 (2.27Å), DT 10 (2.28Å), DC 111 (2.00Å)<br>**Salt bridge:**<br>None<br>**Pi-Pi stacking:**<br>DT 10 (3.63Å)<br>**Pi-cation:**<br>None	−10.163	−76.7022
**14a**	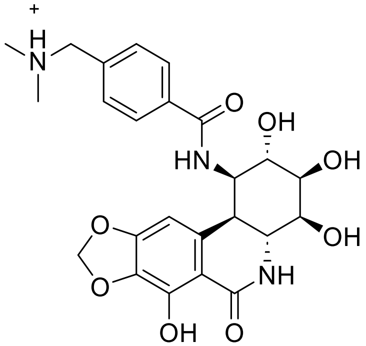	**H-bond:**<br>ASN 352 (1.98Å), TYR 426 (2.71Å), DA 113 (1.86Å and 2.11Å)<br>**Salt bridge:**<br>None<br>**Pi-Pi stacking:**<br>None<br>**Pi-cation:**<br>DT 10 (4.17Å)	−9.64	−52.49
**14b**	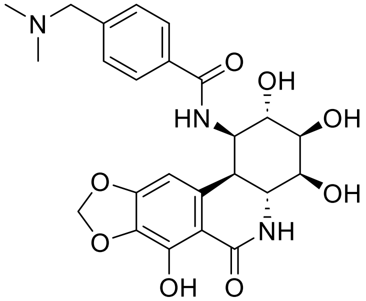	**H-bond:**<br>ASN 352 (1.79Å), TYR 426 (1.94Å)<br>**Salt bridge:**<br>None<br>**Pi-Pi stacking:**<br>None<br>**Pi-cation:**<br>None	−6.915	−37.25

**15a**	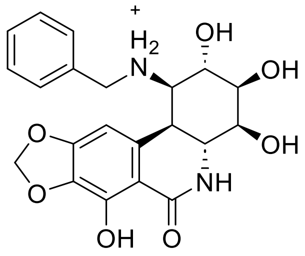	**H-bond:**<br>ASN 352 (2.70Å), LYS 436 (1.80Å), DT 10 (2.37Å)<br>**Salt bridge:**<br>None<br>**Pi-Pi stacking:**<br>DT 10 (3.92Å)<br>**Pi-cation:**<br>None	−6.600	−35.89
**15b**	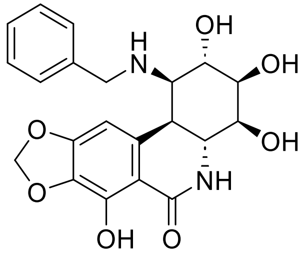	**H-bond:**<br>GLU 356 (1.82Å), TYR 426 (1.60Å and 1.74Å)<br>**Salt bridge:**<br>None<br>**Pi-Pi stacking:**<br>DA 113 (3.51Å and 3.90Å)<br>**Pi-cation:**<br>None	−6.788	−31.98
**15c**	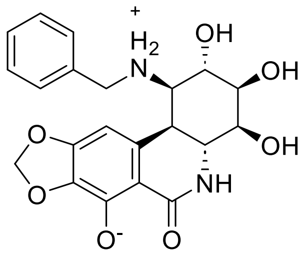	**H-bond:**<br>GLU 356 (1.77Å), TYR 426 (1.59Å and 1.78Å)<br>**Salt bridge:**<br>GLU 356 (4.95Å)<br>**Pi-Pi stacking:**<br>DA 113 (3.53Å and 3.75Å)<br>**Pi-cation:**<br>None	−7.452	−31.12
**16a**	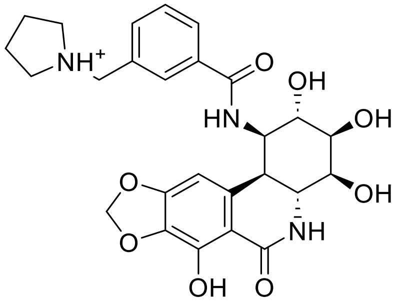	**H-bond:**<br>TYR 426 (1.86Å and 2.64Å)<br>**Salt bridge:**<br>GLU 356 (3.64Å)<br>**Pi-Pi stacking:**<br>DC 112 (4.03Å)<br>**Pi-cation:**<br>None	−8.888	−49.82

**16b**	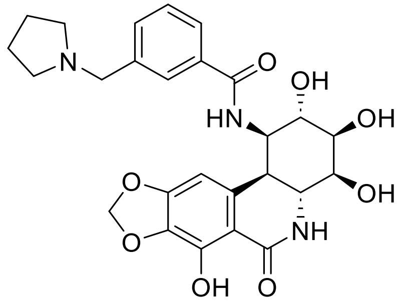	**H-bond:** GLU 356 (2.11Å), TYR 426 (2.40Å), DA 113 (2.00Å and 2.37Å) **Salt bridge:** None **Pi-Pi stacking:** None **Pi-cation:** None	−6.880	−36.30
**17a**	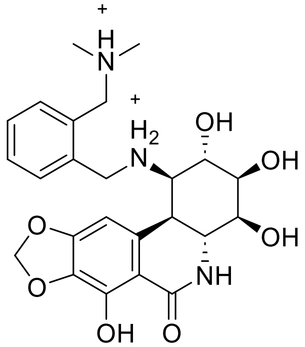	**H-bond:** ALA 351 (2.11Å), GLU 356 (1.75Å) **Salt bridge:** GLU 356 (2.69Å) **Pi-Pi stacking:** DC 112 (3.98Å), DA 113 (4.30Å) **Pi-cation:** LYS 425 (5.17Å)	−8.693	−46.49
**17b**	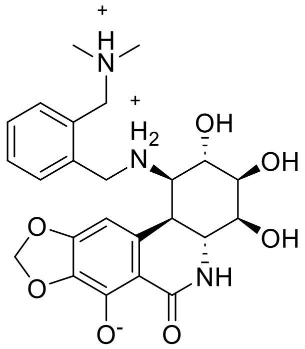	**H-bond:** ASN 352 (2.74Å), GLU 356 (1.73Å), TYR 426 (2.08Å) **Salt bridge:** GLU 356 (2.65Å) **Pi-Pi stacking:** DC 112 (3.56Å), DA 113 (4.38Å) **Pi-cation:** LYS 425 (5.32Å)	−8.023	−34.60

**17c**	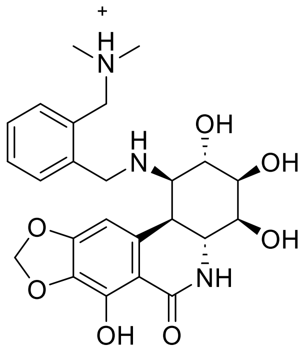	**H-bond:** GLU 356 (1.60Å), LYS 425 (1.85Å), DT 9 (2.60Å), DA 113 (1.97Å) **Salt bridge:** None **Pi-Pi stacking:** None **Pi-cation:** None	−6.462	−50.45
**17d**	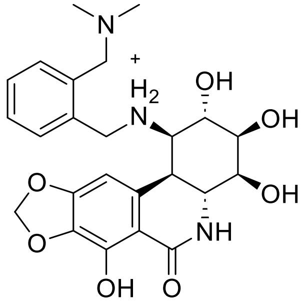	**H-bond:** ASN 352 (1.79Å), MET 428 (2.78Å), LYS 436 (2.06Å) **Salt bridge:** LYS 436 (2.86Å) **Pi-Pi stacking:** DT 10 (3.96Å) **Pi-cation:** None	−6.344	−18.59
**18a**	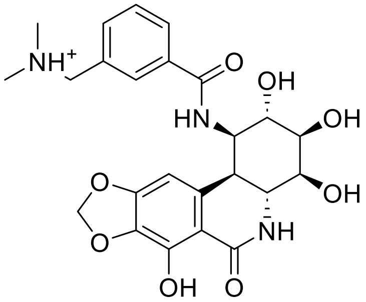	**H-bond:** GLU 356 (2.04Å), TYR 426 (1.95Å and 2.72Å), MET 428 (2.64Å), **Salt bridge:** GLU 356 (2.86Å) **Pi-Pi stacking:** DC 8 (5.44Å), DC 112 (4.14Å), DC 113 (4.12Å), **Pi-cation:** None	−9.167	−48.42

**18b**	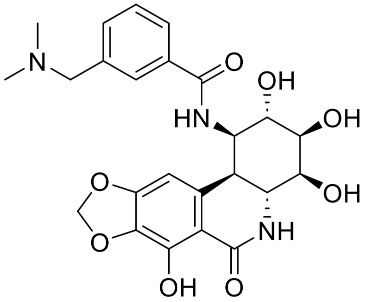	**<u>H-bond:</u>**<br>DT 10 (2.53Å)<br>**<u>Salt bridge:</u>**<br>None<br>**<u>Pi-Pi stacking:</u>**<br>DT 10 (3.88Å and 4.01Å)<br>**<u>Pi-cation:</u>**<br>None	−5.811	−51.04
**19**	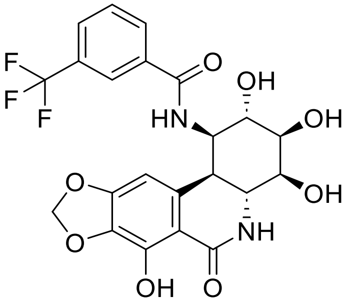	**<u>H-bond:</u>**<br>ASN 352 (2.35Å)<br>**<u>Salt bridge:</u>**<br>None<br>**<u>Pi-Pi stacking:</u>**<br>DT 10 (3.79Å and 3.86Å)<br>**<u>Pi-cation:</u>**<br>None	−7.052	−47.15
**20**	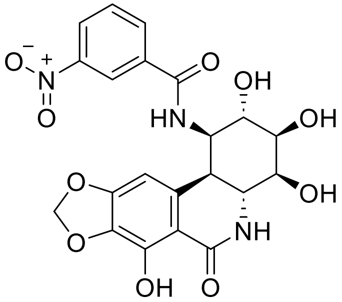	**<u>H-bond:</u>**<br>TYR 426 (2.25Å), ASN 352 (1.82Å), DA 113 (2.13Å)<br>**<u>Salt bridge:</u>**<br>None<br>**<u>Pi-Pi stacking:</u>**<br>None<br>**<u>Pi-cation:</u>**<br>DT 10 (3.69Å)	−8.934	−53.38

Table S2: ADMET and drug-like properties determined by Qikprop module of Schrödinger

molecule	#stars	#amine	#amide	#rotor	#rtvFG	MWt	SASA	FOSA	FISA	PISA	QPlog Po/w	CIQPlogS	QPlog HERG	QPP Caco	QPlog BB	QPPMDCK	#metab	QPlog Khsa	Percent Human Oral Absorption	Rule Of Five	Rule Of Three
**CPT**	0	0	0	2	0	348.3	576.73	186.5	141.9	248.28	1.74	–4.2	–5.076	446.6	–0.854	207.007	3	–0.15	84.575	0	0
**PST**	0	0	0	5	1	325.2	489.39	160.3	275.2	53.78	–1.518	–2.4	–3.652	24.2	–2.082	8.898	6	–0.79	29.896	1	0
**7-Deoxypancratistatin**	0	0	0	4	1	309.2	461.76	160.2	222.1	79.29	–1.261	–2.0	–3.285	77.4	–1.447	31.142	5	–0.72	53.369	0	0
**Narciclasine**	0	0	0	4	1	307.2	470.50	142.4	244.7	83.25	–0.8	–2.7	–3.553	47.2	–1.694	18.278	5	–0.64	52.235	0	0
***trans*-Dihydronarciclasine**	0	0	0	4	1	309.2	468.40	179.4	233.2	55.74	–0.713	–2.6	–3.299	60.8	–1.566	24.016	5	–0.62	54.709	0	0
**7-Deoxy-*trans*-dihydronarciclasine**	0	0	0	3	1	293.2	455.46	179.8	193.3	82.21	–0.582	–2.3	–3.291	145.2	–1.155	61.468	4	–0.62	62.233	0	0
**Lycoricidine**	0	0	0	3	1	291.2	456.64	141.6	204.4	110.53	–0.669	–2.4	–3.537	113.9	–1.268	47.29	4	–0.64	59.839	0	0
**Lycorine**	0	1	0	2	1	287.3	463.91	260.8	86.19	116.86	0.698	–1.9	–4.096	376.1	0.164	190.23	6	–0.36	77.125	0	0
**1**	0	0	0	6	2	429.3	626.61	142.0	263.5	221.02	0.264	–4.5	–5.085	31.4	–2.255	11.743	5	–0.44	55.286	0	0
**2**	0	0	0	4	1	452.4	670.57	146.1	263.3	261.12	0.462	–5.0	–5.691	31.5	–2.289	11.799	5	–0.34	43.518	1	0
**3**	0	0	1	6	1	443.4	678.04	149.4	274.9	253.66	–0.422	–3.9	–4.482	16.7	–2.609	8.969	6	–0.77	20.45	2	1
**4**	0	0	0	6	1	428.3	632.75	143.5	267.1	222.05	–0.019	–4.2	–5.173	28.9	–2.32	10.773	5	–0.51	40.051	1	0
**5**	0	1	0	12	1	527.5	816.35	413.3	258.3	144.7	0.879	–4.4	–6.321	8.7	–2.51	3.273	7	–0.28	10.097	3	2
**6**	0	1	0	11	1	513.5	812.76	380.0	283.5	149.14	0.356	–4.1	–6.595	5.0	–2.841	1.803	7	–0.39	2.747	3	2
**7**	0	1	0	9	1	497.5	733.14	311.3	260.7	161.11	0.023	–3.6	–5.958	8.3	–2.172	3.095	7	–0.43	17.639	2	2
**8**	10	4	0	21	1	642.7	1050.7	562.1	338.2	150.38	–1.192	–1.6	–9.699	0.09	–4.266	0.03	11	–0.76	0	3	2
**9**	10	3	0	21	1	641.7	1062.0	582.1	336.8	143.04	0.155	–3.2	–8.992	0.098	–4.289	0.031	10	–0.44	0	3	2
**10**	9	4	0	21	1	698.8	1119.1	699.1	285.1	134.84	0.184	–2.2	–9.649	0.30	–3.597	0.106	10	–0.42	0	3	2
**11**	0	0	0	7	2	443.4	671.94	147.3	284.6	239.87	0.446	–4.7	–5.636	19.7	–2.702	7.123	5	–0.40	52.755	0	1
**12**	0	1	0	7	1	509.5	707.05	352.4	204.3	150.20	1.198	–4.70	–5.359	28.4	–1.334	11.693	6	–0.06	47.037	1	0
**13**	9	4	0	20	1	640.7	1095.0	634.3	296.8	163.86	0.135	–2.2	–10.05	0.23	–3.873	0.08	10	–0.34	0	3	2
**14**	0	1	0	8	1	485.4	745.83	323.3	269.5	152.86	–0.219	–3.5	–6.238	6.8	–2.335	2.509	7	–0.50	14.716	2	2
**15**	0	1	0	7	1	414.4	629.39	166.7	225.6	237.03	0.033	–3.29	–5.977	17.9	–1.592	7.081	7	–0.45	36.606	1	2

**16**	0	1	0	8	1	511.5	688.84	277.7	261.3	149.78	-0.114	–4.06	–5.254	8.2	–1.881	3.051	7	–0.40	3.774	3	2
**17**	1	2	0	9	1	471.5	649.33	270.6	242.9	135.73	-0.632	–2.59	–5.69	3.06	–1.308	1.161	9	–0.46	18.987	1	2
**18**	0	1	0	8	1	485.4	720.91	304.6	264.1	152.17	-0.169	–3.54	–5.836	7.7	–2.115	2.855	7	–0.47	15.937	2	2
**19**	0	0	0	6	1	496.3	686.09	143.1	264.7	160.98	0.895	–5.5	–5.217	30.5	–2.137	50.118	6	–0.35	45.817	1	0
**20**	3	0	0	7	1	473.3	671.21	143.4	361.4	166.32	-0.684	–4.72	–5.131	3.6	–3.385	1.163	6	–0.55	7.192	2	1

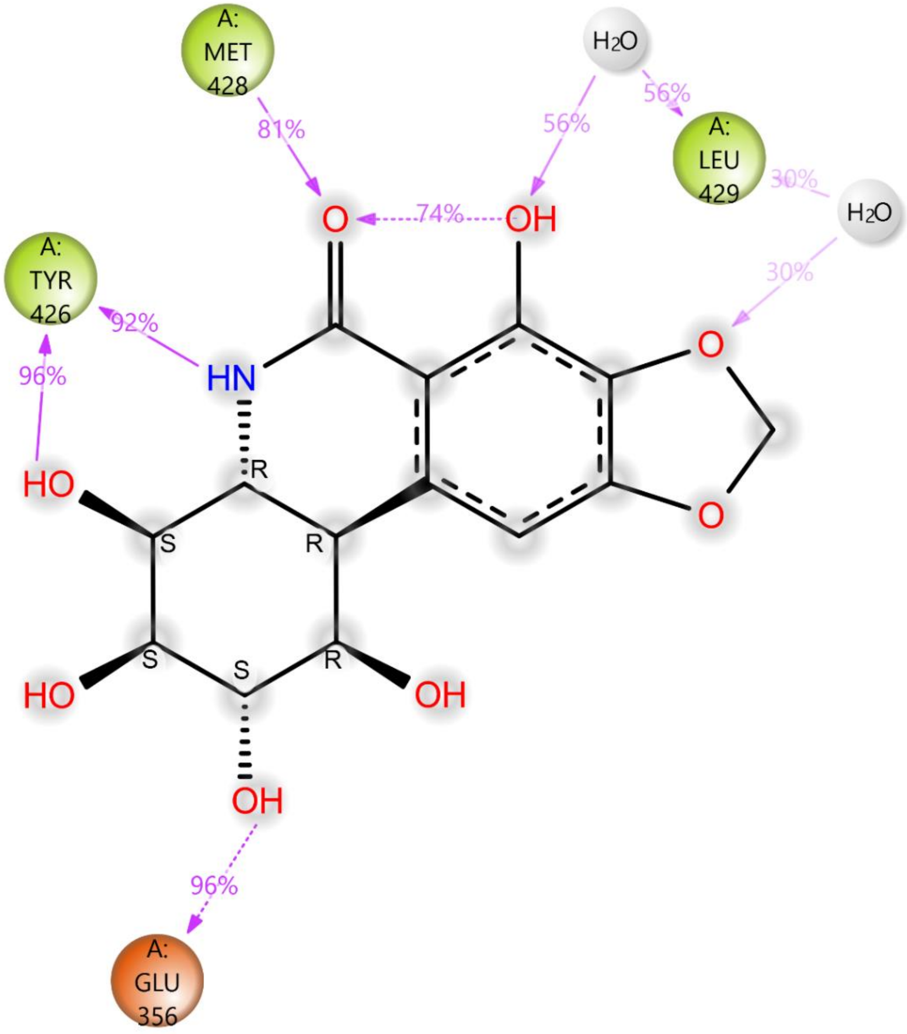

Figure S1. A diagram illustrating the 2D protein-ligand contact interactions of PST with Topo I (PDB: 1T8I) throughout the simulation period. H-bonds are depicted in magenta.

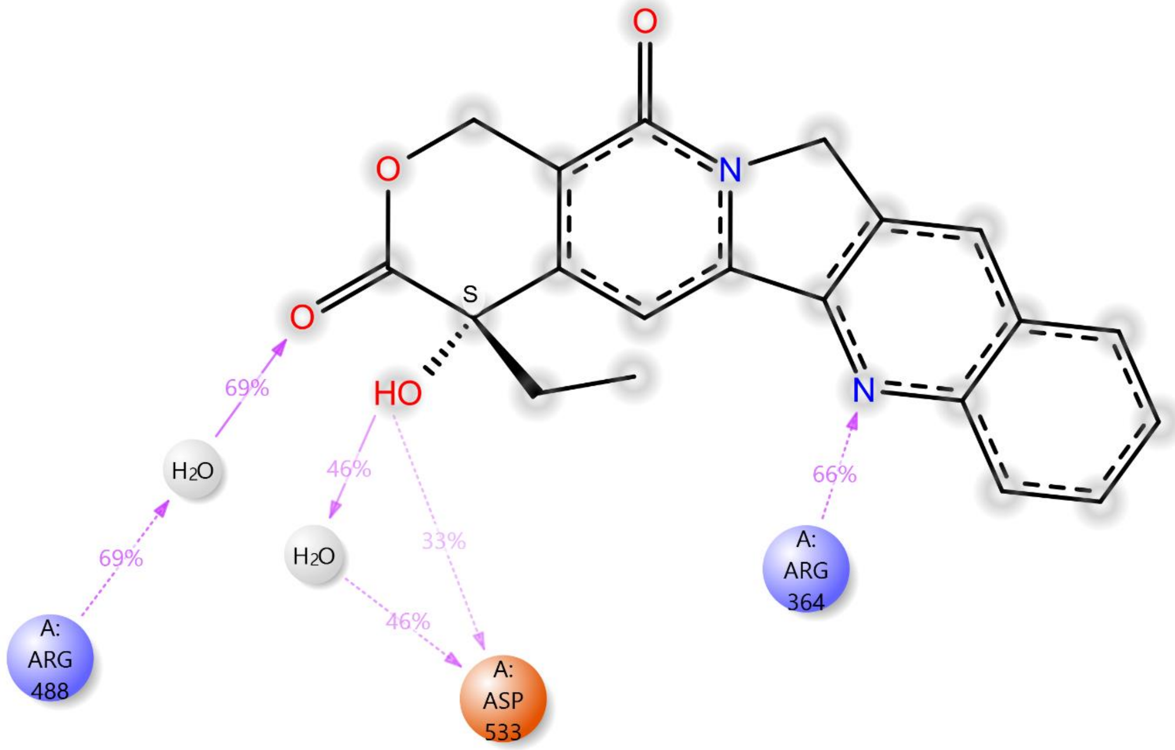

Figure S2. A diagram illustrating the 2D protein-ligand contact interactions of CPT with Topo I (PDB: 1T8I) throughout the simulation period. H-bonds are depicted in magenta.

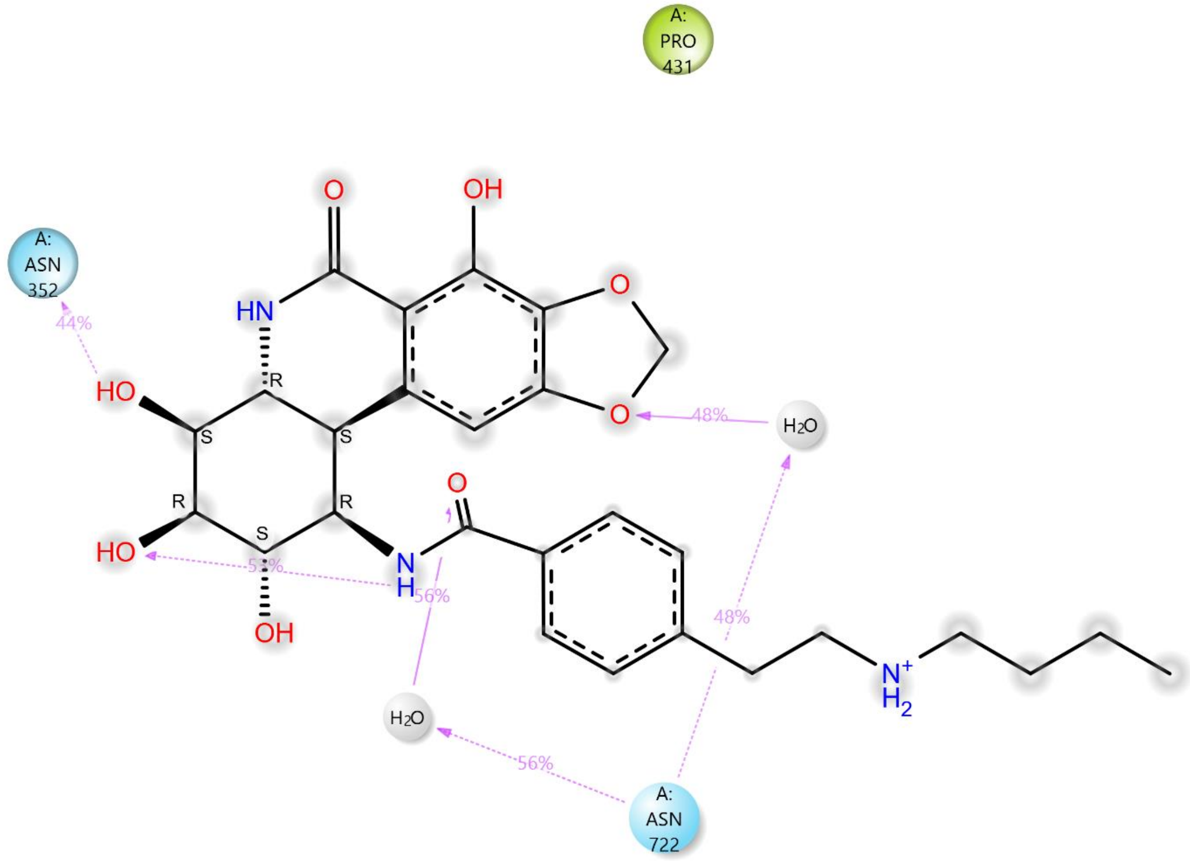

Figure S3. A diagram illustrating the 2D protein-ligand contact interactions of compound **5** with Topo I (PDB: 1T8I) throughout the simulation period. H-bonds are depicted in magenta.

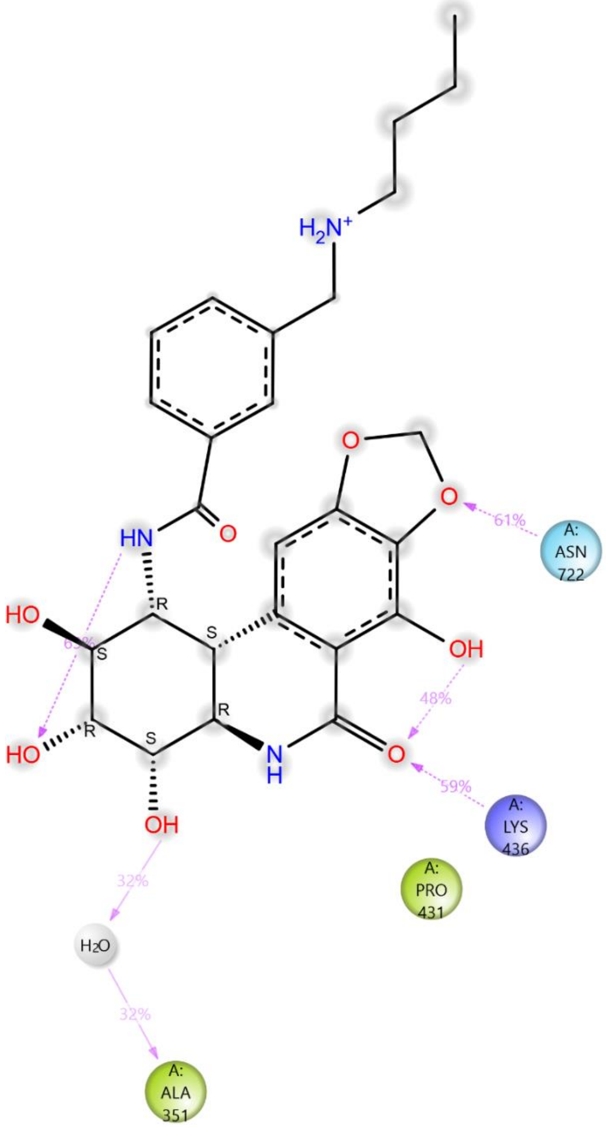

Figure S4. A diagram illustrating the 2D protein-ligand contact interactions of compound **6a** with Topo I (PDB: 1T8I) throughout the simulation period. H-bonds are depicted in magenta.

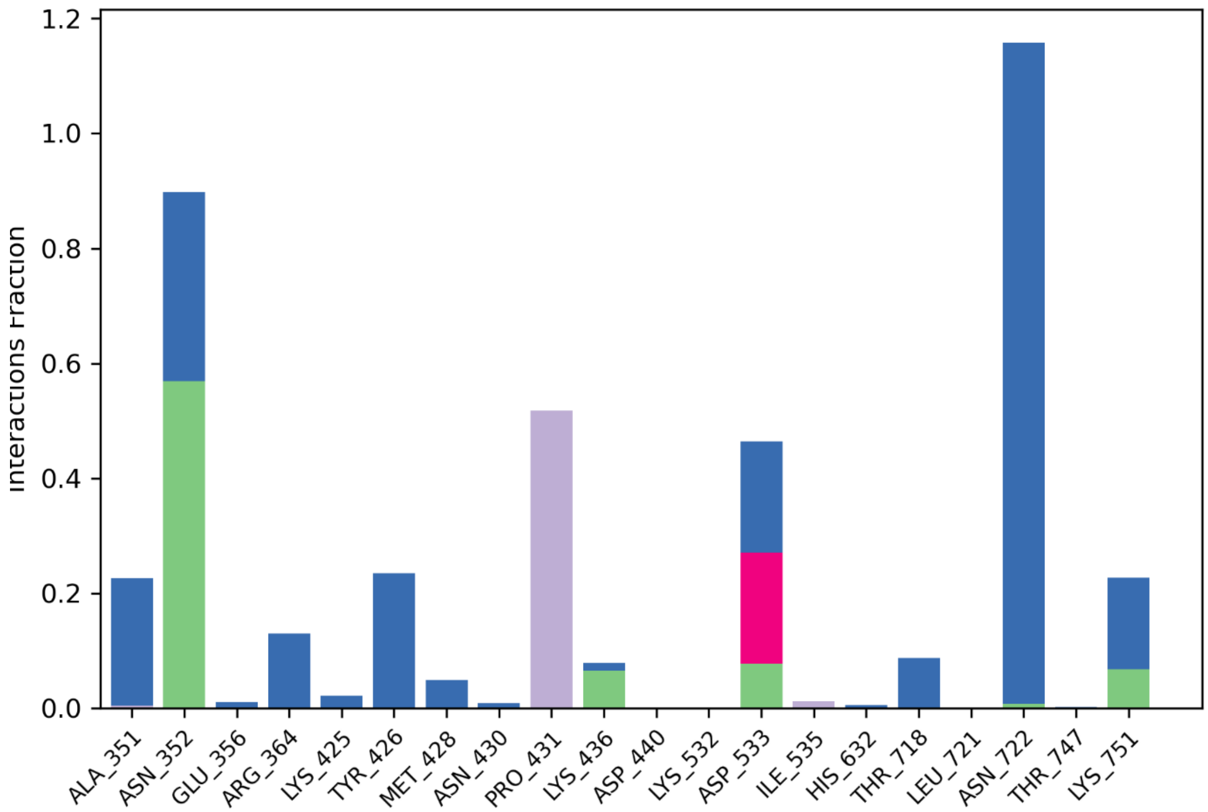

Figure S5. A histogram illustrating the binding mode of compound **5** with Topo I (PDB: 1T8I) throughout the simulation run. The different interactions are color-coded: H-bonds in green, hydrophobic interactions in grey, ionic bonds in deep pink, and water bridges in blue.

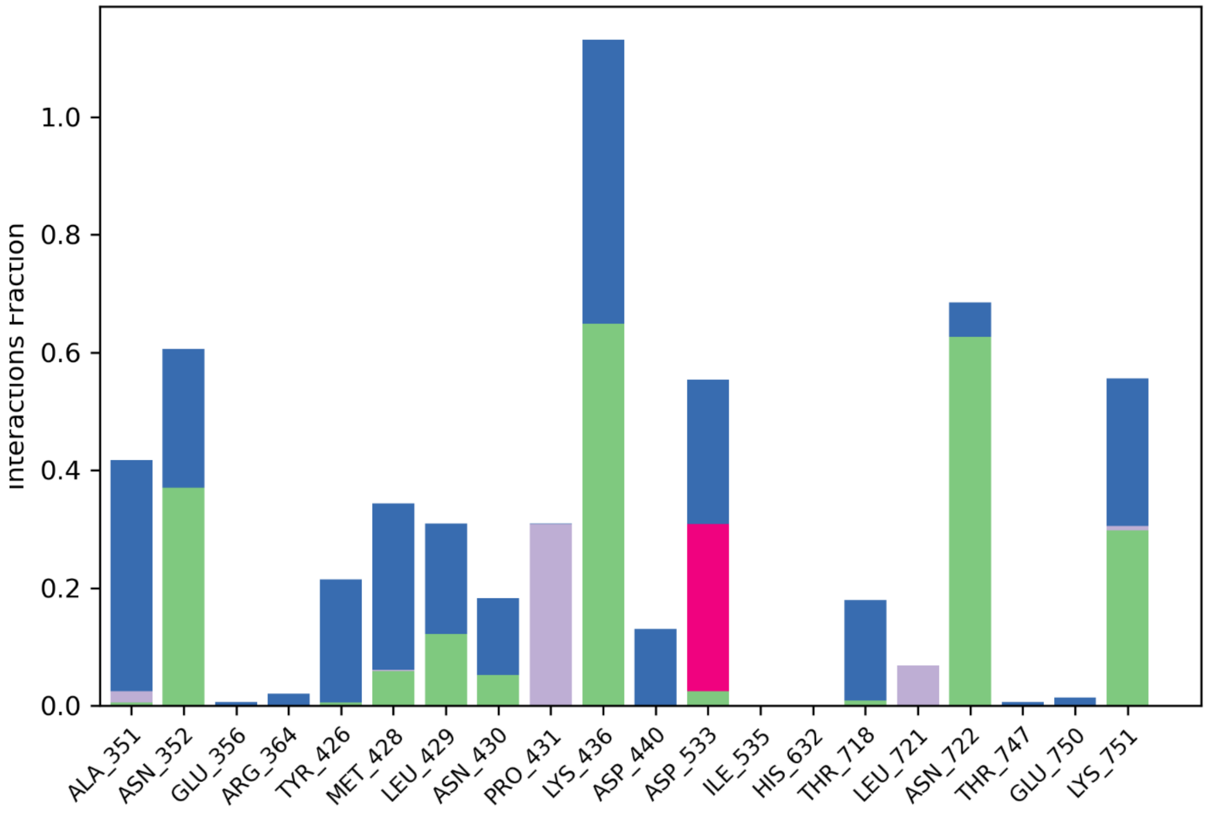

Figure S6. A histogram illustrating the binding mode of compound **6a** with Topo I (PDB: 1T8I) throughout the simulation run. The different interactions are color-coded: H-bonds in green, hydrophobic interactions in grey, ionic bonds in deep pink, and water bridges in blue.
